# Identification of Critical Transcriptomic Signaling Pathways in Patients with H Syndrome and Rosai-Dorfman Disease

**DOI:** 10.1007/s10875-020-00932-1

**Published:** 2020-12-07

**Authors:** Samuel Lara-Reyna, James A. Poulter, Elton J.R. Vasconcelos, Mark Kacar, Michael F. McDermott, Reuben Tooze, Rainer Doffinger, Sinisa Savic

**Affiliations:** 1 Leeds Institute of Rheumatic and Musculoskeletal Medicine, University of Leeds, Leeds, LS9 7TF UK; 2 Leeds Institute of Medical Research, University of Leeds, Leeds, LS9 7TF UK; 3 Leeds Omics, University of Leeds, Leeds, LS2 9JT UK; 4 Department of Clinical Immunology and Allergy, St James’s University Hospital, Leeds, LS9 7TF UK; 5 Section of Experimental Haematology, Leeds Institute of Cancer and Pathology, University of Leeds, Leeds, UK; 6 Department of Clinical Biochemistry and Immunology, Addenbrooke’s Hospital, Cambridge, CB2 2QQ UK

**Keywords:** H syndrome, systemic autoinflammatory disease, interferon gamma

## Abstract

Biallelic mutations in *SLC29A3* cause histiocytosis-lymphadenopathy plus syndrome, also known as H syndrome (HS). HS is a complex disorder, with ~ 25% of patients developing autoinflammatory complications consisting of unexplained fevers, persistently elevated inflammatory markers, and unusual lymphadenopathies, with infiltrating CD68^+^, S100^+^, and CD1a^−^ histiocytes, resembling the immunophenotype found in Rosai-Dorfman disease (RDD)**.** We investigated the transcriptomic profiles of monocytes, non-activated (M0), classically activated (M1), and alternatively activated macrophages (M2) in two patients with HS, one without autoinflammatory (HS1) and one with autoinflammatory complications (HS2). RNA sequencing revealed a dysregulated transcriptomic profile in both HS patients compared to healthy controls (HC). HS2, when compared to HS1, had several differentially expressed genes, including genes associated with lymphocytic-histiocytic predominance (e.g. *NINL*) and chronic immune activation (e.g. *B2M*). The transcriptomic and cytokine profiles of HS patients were comparable to patients with SAID with high levels of TNF. *SERPINA1* gene expression was found to be upregulated in all patients studied. Moreover, higher levels of IFNγ were found in the serum of both HS patients when compared to HC. Gene ontology (GO) enrichment analysis of the DEGs in HS patients revealed the terms “type I IFN,” “IFNγ signaling pathway,” and “immune responses” as the top 3 most significant terms for monocytes. Gene expression analysis of lymph node biopsies from sporadic and H syndrome-associated RDD suggests common underlying pathological process**.** In conclusion, monocytes and macrophages from both HS patients showed transcriptomic profiles similar to SAIDs and also uniquely upregulated IFNγ signature. These findings may help find better therapeutic options for this rare disorder.

**Supplementary Information:**

The online version contains supplementary material available at 10.1007/s10875-020-00932-1.

## Introduction

Biallelic mutations in *SLC29A3*, encoding the intracellular equilibrate nucleoside transporter 3 (ENT3), cause a range of related genetic disorders, collectively known as histiocytosis-lymphadenopathy plus (OMM #602782) or H syndrome (HS) [1–3]. The spectrum of clinical features associated with HS includes dermatological (hyperpigmentation and hypertrichosis) and systemic manifestations, such as hepatosplenomegaly, hearing loss, hypogonadism, heart anomalies, short stature, hyperglycemia (non-autoimmune insulin-dependent diabetes mellitus (IDDM)) and camptodactyly [2, 4]. The histiocytosis, when present, most closely resembles Rosai-Dorfman disease (RDD), which is characterized by infiltrating CD68^+^, S100^+^, and CD1a^−^ histiocytes [5]. To date, no correlation between mutation type, or genomic location, and severity of the phenotype has been demonstrated [2].

Recently, HS has been described as a systemic autoinflammatory disease (SAID), since up to 25% of HS patients develop typical complications including unexplained fevers, seronegative arthritis, and persistently elevated inflammatory markers [2, 4, 6, 7]. The term “autoinflammation” was coined in 1999 to describe a non-infectious inflammatory state, which was due to disturbance of the innate immune system with monocytes, macrophages, and neutrophils being critical cellular mediators of this process [8]. The wide range of clinical manifestations in HS is probably due to the fact that ENT3 plays an essential role in several biochemical reactions, which include the regulation of nucleic acids, lysosomal homeostasis, mitochondrial function, and cellular migration [9–11]. Considering that mitochondrial dysfunction and oxidative stress are closely linked with several critical innate-immune inflammatory pathways, including NLRP3 inflammasome activation, and the fact that macrophages show the highest expression of this transporter [9], this might explain why some patients develop SAID-like complications.

Murine studies have shown that macrophages have a critical role in the disease pathogenesis. Similar to the clinical features observed in patients, Ent3^−/−^ mice also develop spontaneous splenomegaly and lymphadenopathy early in life, with a significantly higher number of macrophages in the spleen but typical numbers of T and B cells [9]. Furthermore, in these mice, splenic macrophages show increased M-CSFR expression with increased levels of M-CSF in the sera of these mice [9]. While blocking M-CSF resulted in a partial reduction of the number of splenic macrophages, it did not change the mortality rate seen in Ent3^−/−^ mice or completely rescue the phenotype, indicating that other mechanisms must be responsible for this phenotype.

To investigate which biochemical processes and inflammatory pathways are most relevant to the pathogenesis of inflammatory complications in HS, we performed transcriptomic analyses in monocytes, non-activated macrophages (M0), classically activated macrophages (M1), and alternatively activated macrophages (M2) from two HS patients, one without and one with inflammatory complications. We compared the findings to a similar analysis performed in two patients with SAID. Moreover, we compared our transcriptomic data to other published cases of classical SAIDs. Finally, we compared the transcriptomic profile of the HS patient with autoinflammatory complications, with tissue biopsies (RDD) obtained from sporadic cases and genetically confirmed HS patients. This study provides the first transcriptomic insights into the dysregulated cellular mechanisms in monocytes and macrophages from HS patients. We show that monocytes and macrophages share similar transcriptomic and cytokine profiles as SAIDs.

## Methods

### Patients’ Characteristics and Study Design

Patient blood samples used for this study were obtained with ethics approval (REC 10/H1306/88, National Research Ethics Committee Yorkshire and Humber–Leeds East), and all the studies involving human samples from healthy control (HC) volunteers were approved by the Health Research Authority (REC reference 17/YH/0084). Patients were recruited from the Department of Clinical Immunology and Allergy, St James’s University Hospitals, Leeds, UK. Informed written consent was obtained from all participants at the time of the sample collection. Age- and sex-matched HC were recruited from the St James’s University Hospitals, Leeds, UK. Details for the tissue biopsies can be found in Supplementary Table 1.

### Preparation of Human Blood and Isolation of Immune Cells

Blood samples were collected in EDTA precoated tubes and processed within the next 4 h. Peripheral blood mononuclear cells (PBMCs) were isolated from whole blood using a standard density gradient centrifugation method. Blood was mixed with an equal volume of DPBS (without Ca^2+^ and Mg^2+^, containing 2% heat-inactivated fetal bovine serum (FBS)) and carefully layered onto Lymphoprep (StemCell) and centrifuged at 1100×*g* for 20 min without brakes. The white buffy layer was collected and washed twice in DPBS (2% FBS) by centrifuging at 150×*g* for 10 min without brakes, to remove platelets. After PBMCs were obtained, CD14^+^ monocytes were isolated by immunomagnetic negative selection, using the EasySep Human Monocyte Isolation Kit (StemCell). Isolated monocytes were immediately lysed with TRIzol or differentiated to macrophages as described in the next sections. Table 1

**Table 1:** Patients’ and HC demographics

Subject	Age	Sex	Mutation
H syndrome 1 (HS1)	18	F	*SLC29A3* NM_018344.5:c.182G > T, p.(Gly61Val)
H syndrome 2 (HS2)	26	F	*SLC29A3* NM_018344.5:c.300 + 1G > A
Undefined SAID (uSAID)	55	M	*TNFRSF1A* c.1328G > T, p(Gly443Val)
TRAPS	31	F	*TNFRSF1A* c.236C > T (p.Thr79Met)
HC1	36	F	N/A
HC2	45	M	N/A
HC3	26	F	N/A
HC4	18	F	N/A
HC5	35	F	N/A
HC6	36	F	N/A
HC7	27	F	N/A
HC8	22	M	N/A
HC9	44	F	N/A

### M1/M2 Macrophage Differentiation and Polarization

Isolated human monocytes were cultured in complete RPMI medium (10% FBS, 50 U/ml penicillin, 50 μg/ml streptomycin and 1% L-glutamine) (Merck) supplemented with 20 ng/mL human GM-CSF (PeproTech) for macrophage differentiation and incubated for 6 days, adding fresh media on day 3. On day 6, M0 macrophages were activated with 100 ng/mL human IFN-γ (PeproTech) and 50 ng/mL LPS, for M1 macrophage polarization, or 20 ng/mL IL-13 (PeproTech) and IL-4 (PeproTech), for M2 macrophage polarization, and incubated for 24 h. Monocytes were initially seeded at a density of 0.5 × 10^6^ and cultured in tissue culture-treated 12 well plates.

### Flow Cytometry

Characterization of the macrophages was done through flow cytometry. On day 7, cells were washed twice with DBPS and detached using DPBS with EDTA 10 mM. Cells were washed with DPBS and resuspended in brilliant stain buffer (BSB) with human and mouse serum for 20 min on ice. Cells were stained with the surface markers for M1 (CD64^+^, CD80^+^, and CD86^+^) and M2 (CD64^+^, CD206^+,^ and CD209^+^) for 30 min on ice. Finally, cells were resuspended in BSB in FACS collection tubes, and samples were run in the CytoFLEX-LS (Beckman Coulter). All antibodies used are listed in detail in the table of regents.

### RNA Preparation and Analysis

Total RNA was obtained by using TRIzol and Phasemaker Tubes (Thermo Fisher Scientific) according to the manufacturers’ protocol. RNA quality and quantity were further determined by NanoDrop spectrophotometer and with the Agilent TapeStation (Agilent Technologies).

### RNA Sequencing

Library preparation and RNA sequencing (RNA-seq) were performed on an Illumina Novaseq and generating 150 bp paired-ended reads (Novogene Bioinformatics Technology Co., LTD). An average of 55.0 million raw reads were generated per sample (effective rate average 97.6% and Q30 average of 94.5%). RNA-seq raw reads were trimmed using TrimGalore [12] to remove library adaptors sequences and low-quality reads (QV ≤ 30). High-quality reads were mapped to the reference genome (GRCh38/hg38) using STAR [13], and then the Cufflinks-Cuffdiff (v2.2.1) pipeline [14] was employed to perform transcriptome assembly, normalization, and differential expression (DE) analyses. Monocytes and macrophages showed an average of 89.3% uniquely mapped reads, whereas tissue samples showed an average of 48.5%. Processed and raw RNA-seq data have been deposited to the NCBI Gene Expression Omnibus (GEO) database (accession number: GSE155697). Gene-ontology (GO) enrichment analysis of the DEGs was performed using the DAVID (v6.8) functional annotation analysis tool [15].

### Cytokine Detection

The supernatant from cultured macrophages and patients’ serum were frozen and stored at − 80 °C. TNF, IL-12, IL-6, IL-1β, IFNγ, and IL-2 (R+D Systems Fluorokinemap) cytokines were measured by using the multiplex Luminex analyzer (Bio-Plex, Bio-Rad, UK). Serum was collected immediately after the sample collection, and supernatants from macrophages were collected before processing the cells.

### Quantification and Statistical Analysis

Flow cytometry and cytokine analysis were analyzed by multiple independent *t* tests, and statistical significance determined by the Holm-Sidak method. Each parameter was analyzed individually without assuming a consistent SD. GraphPad Prism 8 software was used to do all analyses. *P* values of < 0.05 were statistically significant. Nonparametric tests were used to compare the medians between groups.

### Table of Reagents

Details of all reagents can be found in the Supplementary Table 2.

## Results

### Patients’ Characteristics

#### H Syndrome

The first H syndrome patient (HS1) is an 18-year-old female of Pakistani consanguineous descent. The patient initially presented at the age of 12 with IDDM associated with positive anti-islet cell antibodies. Within a year of initial presentation, the patient developed camptodactyly and fleeting pain and swelling in other joints, including the right knee, wrist, elbow, and temporomandibular joint. Further investigations in pediatric rheumatology did not show any evidence of active inflammatory arthritis, and the joint symptoms settled spontaneously within the following 6 to 9 months. Subsequent genetic analysis revealed a rare, previously unreported, homozygous variant in *SLC29A3* (NM_018344.5:c.182G>T, p.(Gly61Val)) as the likely cause of disease. The patient’s younger sister, who, in addition to camptodactyly and IDDM, also had short stature (9th centile) and hypertrichosis was later found to share the same genetic variant. HS1 has remained otherwise well, apart from IDDM and camptodactyly, and has not developed any inflammatory complications.

The second patient (HS2) is a 26-year-old female of consanguineous Pakistani descent. A detailed description of the patient’s clinical features and treatment history has been published previously [16]. Briefly, the patient first came to medical attention at the age of 2 years after presenting with a self-limiting, well-delineated telangiectatic rash. Over the ensuing decade, the patient developed arthralgia, fatigue and dactylitis, and at the age of 9, the patient was found to have RDD causing non-tender submandibular lymphadenopathy. Subsequently, she was diagnosed with HS due to the identification of a homozygous splice-site mutation in *SLC29A3* (NM_018344.5:c.300+1G>A). The patient’s other clinical features are consistent with this diagnosis, including short stature, IDDM, and pancreatic insufficiency. In the ensuing years, she has developed numerous inflammatory complications, which continue to be problematic, including sporadic fevers, transient synovitis, persistently elevated CRP, and progressive RDD, which now also involves her skin. She was treated with numerous disease-modifying antirheumatic (DMARD) medications, including biological therapies such as rituximab and tocilizumab, which have not been effective.

#### SAID

The first undefined SAID patient (uSAID) is a 55-year-old male who has a life-long history of unexplained fevers associated with flu-like symptoms lasting anywhere between a few days to weeks. The patient’s clinical picture is complicated by recurrent episodes of cellulitis as a result of complex trauma in his left knee. Sequencing revealed a previously unreported heterozygous single nucleotide variant of unknown significance c.1328G>T, p(Gly443Val), in exon 10 of the *TNFRSF1A* gene. The patient is receiving on-demand treatment with prednisolone, which he infrequently takes for the autoinflammatory complications, similar to those presented by patients with TNF-Receptor Associated Periodic Syndrome (TRAPS).

The second patient (TRAPS) is a 31-year-old female who has been symptomatic since her early teens with episodic fevers and serositis. She carries a c.236C>T (p.Thr79Met) mutation in *TNFRSF1A*, previously described as p.Thr50Met [17]. At present, she is treated effectively with anakinra (synthetic IL-1 receptor antagonist).

### Transcriptomic Differences of Monocytes and Macrophages

**Fig. 1:**
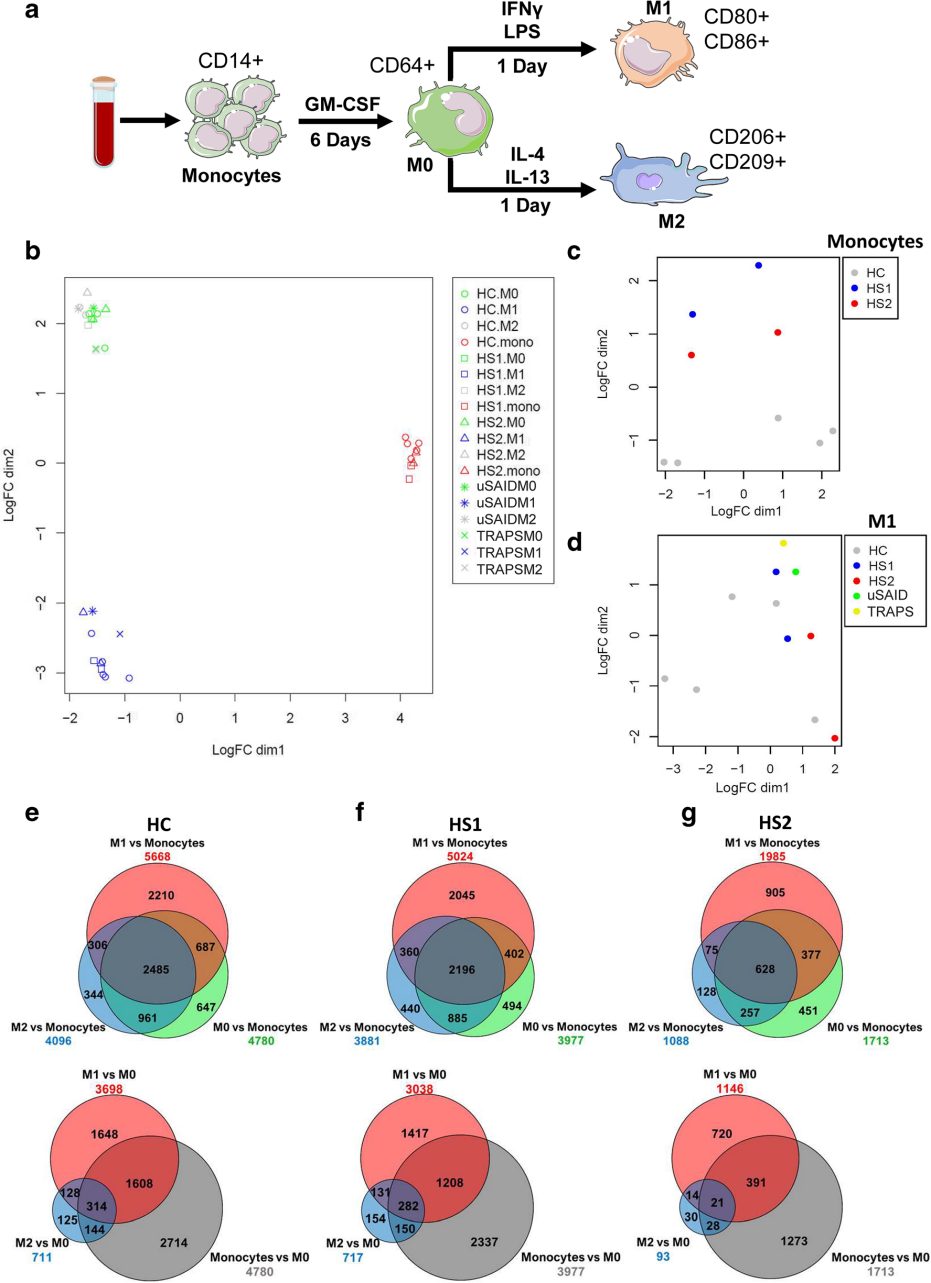
Transcriptomic sample analysis in both monocytes and macrophages. (**a**) CD14^+^ monocytes obtained by negative selection were differentiated into macrophages for 6 days, and then M1 macrophage polarization was accomplished by stimulating with 100 ng/ml human IFNγ and 50 ng/ml LPS. M2 macrophage polarization was accomplished by stimulating with 20 ng/ml IL-13 and 20 ng/ml IL-4. M0 macrophages were cultured for 7 days. (**b**) Multidimensional scaling (MDS) plot showing the global transcriptomic profile of all the HC (1–5) and patients’ samples, where all the different cell types are shown. (**c** and **d**) MDS plot showing the global transcriptomic profile in monocytes and M1 of all the HC and patients’ samples. (e–g) The number of differentially expressed genes (DEGs) in pairwise comparisons of the different cell types are given, where monocytes (top) and M0 (bottom) are compared to each other cell type. The Venn diagrams show shared and unique DEGs for each cell type in (**e**) HC, (**f**) HS1, and (**g**) HS2 samples

To study the transcriptomic profile of monocytes, M0, M1, and M2 macrophages, we obtained CD14^+^ monocytes from 2 HS, 2 SAID patients, and 5 HC and differentiated the cells according to the protocol summarized in Fig. 1a. To determine whether the macrophage differentiation was successful, the global transcriptomic differences between the different cell types, in each group, were assessed and visualized using multidimensional scaling (MDS) plots (Fig. 1b–d). Monocytes, M0, and M1 macrophages were clearly separated, by at least three units on the MDS plot, for each sample (Fig. 1b). Although the M2 and M0 macrophages did not show a distinct global transcriptomic difference by MDS, we found this to be consistent with another published dataset available of M2 differentiated from M0 macrophages [18]. Moreover, expression of the surface markers CD80/CD86 and CD206/CD209 showed high expression in M1 and M2 macrophages, respectively. To further corroborate the differences between the cell types, pairwise comparisons were made between the different cell types in each group (Fig. 1e–g). As shown in the Venn diagrams, a large number of transcripts were found to be differentially expressed in M0, M1, and M2, when compared to monocytes; for example, HC (*n* = 5) and HS1 showed 2210 and 2045 DEGs in M1 macrophages, respectively, and M1 macrophages from patient HS2 presented 905 DEGs (Fig. 1e–g top). When compared to M0 macrophages, the transcriptomic differences of monocytes were more evident, with 2714 (HC), 2337 (HS1), and 1273 (HS2) DEGs (Fig. 1e–g bottom). Similarly, when comparing to monocytes, M2 macrophages showed 344 (HC), 440 (HS1), and 128 (HS2) DEGs in each respective group (Fig. 1e–g top).

The number of DEGs was reduced in the different cell types from the uSAID and TRAPS patients. We observed 80 and 149 unique DEGs in M2 and M1 macrophages from uSAID patient and 102 and 93 in M2 and M1 macrophages from the patient with TRAPS (Supplemental Fig. 1C-D). The remaining comparisons of all the DEGs in each cell type from each sample group are shown in Supplemental Fig. 1E-G.

### Transcriptomic Profile of Two Different H Syndrome Patients

To determine which cellular processes might be responsible for the differences in the inflammatory phenotypes observed between the two HS patients, we first compared the global transcriptomic profile of cells derived from HS patients. While heterogeneity is an important factor that influences the transcriptomic profiles of cell subset in different individuals [19], we still observed a clear degree of separation in the monocytes from the two HS patients when compared to the HC samples (Fig. 1c) and to a lesser degree in the other cell subsets (Fig. 1d; Supplemental Fig. 1A-B).

**Fig. 2:**
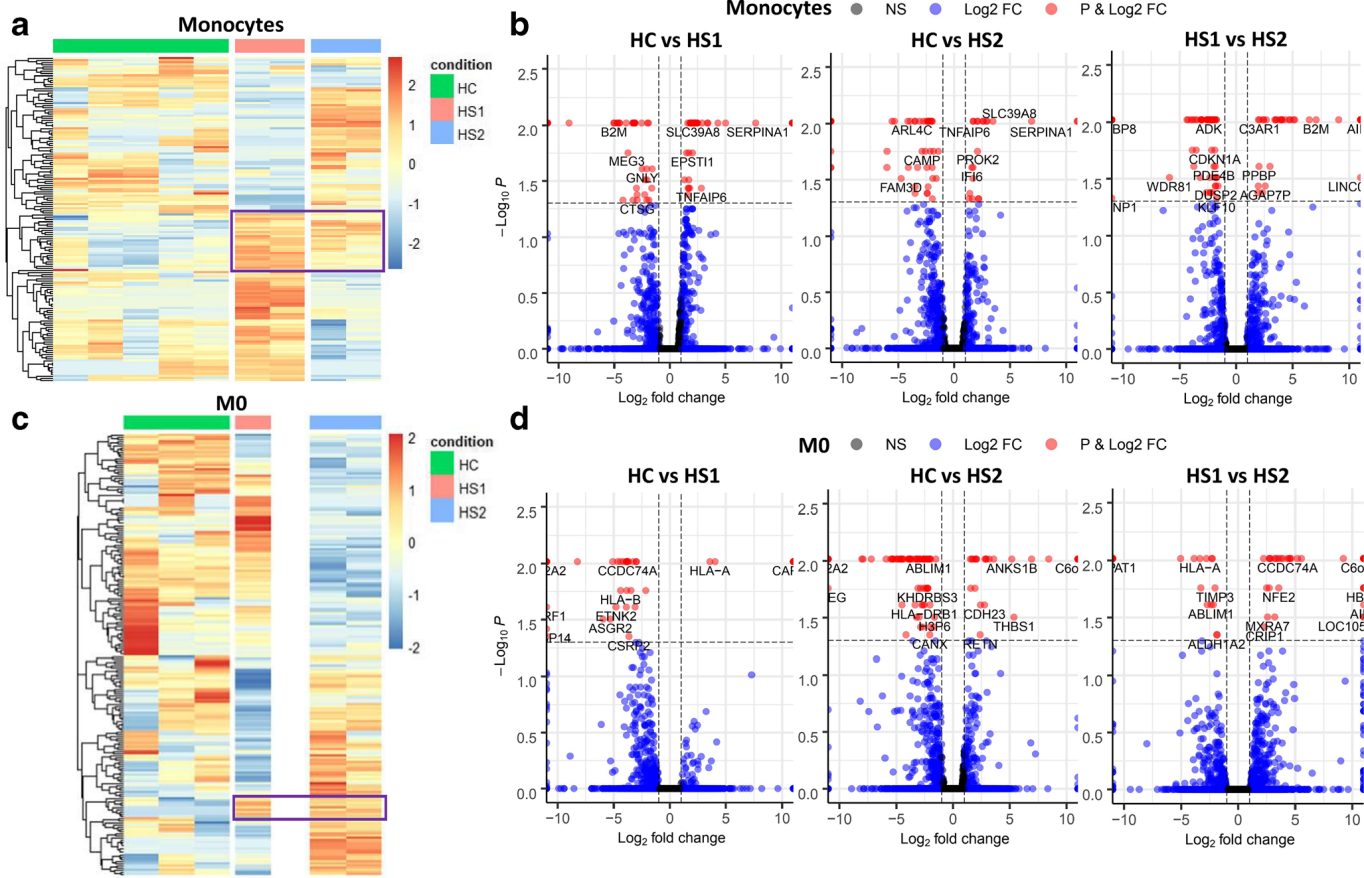

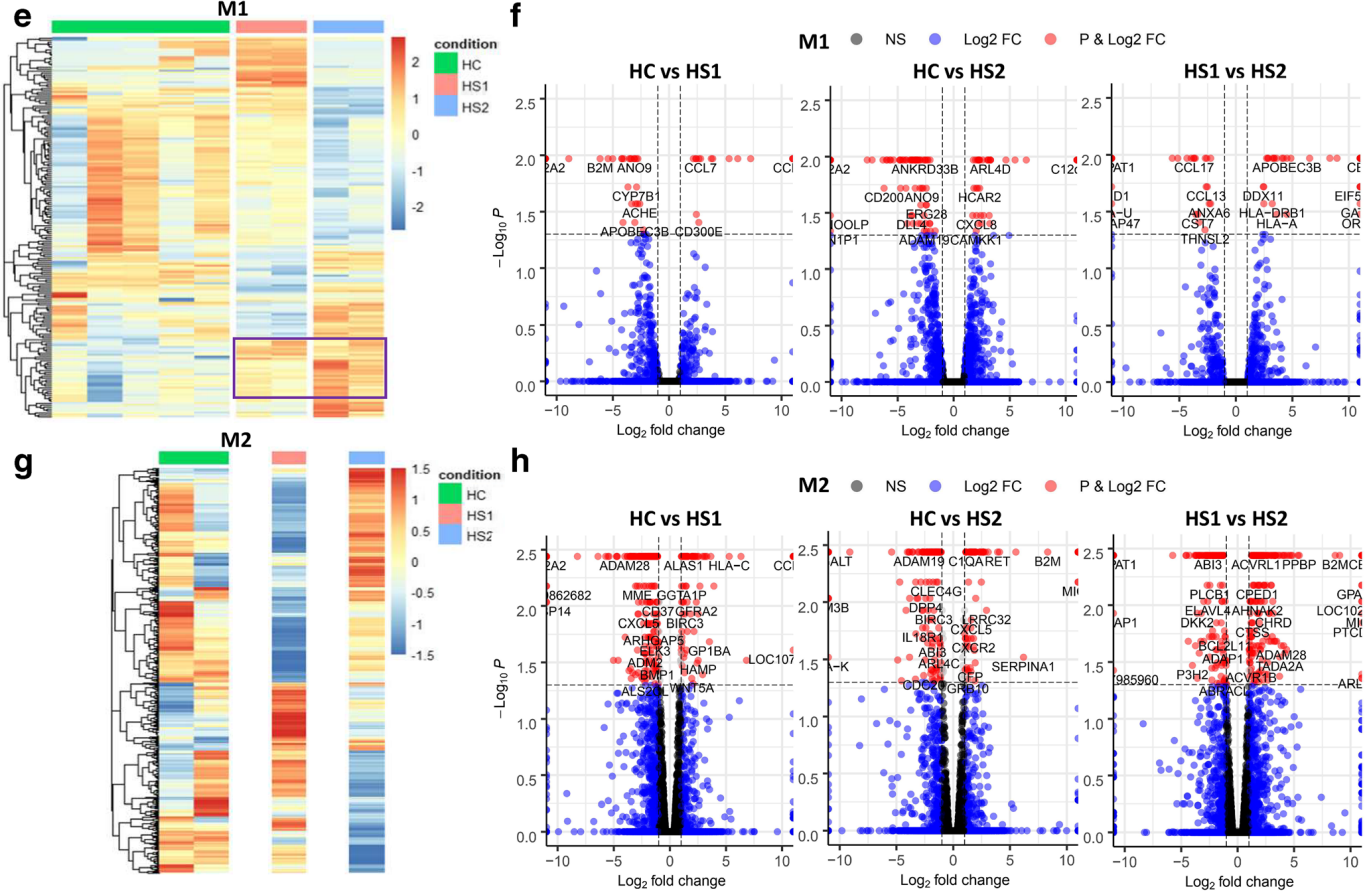
Different transcriptomic profile in HS patients. Pairwise differential expression comparison between HC (1–5) and patients’ samples, as well as between the HS1 and HS2 patients in all different cell types. (**a**, **c**, **e,** and **g**) Heatmaps show only the DEGs (*q* < 0.05) between the 3 different groups, HC, HS1, and HS2, monocytes, M0, M1, and M2, accordingly. The expression was standardized, and the color shading scale represents Log2(FPKM) values. Genes (rows) were hierarchically clustered (Euclidean). (**b**, **d**, **f,** and **h**) Volcano plots showing the DEGs in Log2 (fold change) and *p* value for the comparisons of HC vs HS1 (left), HC vs HS2 (middle), and HS1 vs HS2 (right) in monocytes, M0, M1, and M2, accordingly. Differentially expressed genes in volcano plots (FC ≥ 1, *p* < 0.05 with FDR) are depicted in red. The purple boxes show a cluster of genes similarly expressed in the two HS patients

We next conducted pairwise comparisons of the DEGs between both HS patients, as well as independently against the HC samples, for each cell type. Monocytes from HS patients shared a cluster of upregulated genes when compared to the HC controls (Fig. 2a), and a total of 73 and 70 DEGs were shown in patients HS1 and HS2, respectively (Supplement Table 4). The shared upregulated DEGs in both HS patients included *SERPINA1*, *TNFAIP6,* and *SLC39A8* (Fig. 2b). When monocytes from patients HS1 and HS2 were compared, we found 77 DEGs with several *HLA* genes being overexpressed in patient HS2, as well as *B2M,* which encodes β2-Microglobulin, a component of MHC class I molecules (Fig. 2b).

When comparing transcriptomic profiles of M0 macrophages, patient HS1 showed a profile resembling the HC samples rather than HS2 (Fig. 2c). The number of DEGs in M0 macrophages was 58 in HS1 and 154 in HS2, when compared to HC samples. Similarly, when the two HS patients were compared to each other, we observed 71 DEGs, including *B2M* and *HLA* genes, upregulated and lysosomal-associated membrane protein 3 (*LAMP3*) downregulated in patient HS2 (Fig. 2d).

A cluster of overexpressed genes was found in M1 macrophages from the two HS patients when compared to HC samples (Fig. 2e). In M1 macrophages, several inflammatory genes such as IFN-responsive genes, TNF, IL-1α and IL-1β were upregulated in both HS patients (Fig. 2f). In M2 macrophages, we still observed a different transcriptomic profile in the two HS patients (Fig. 2g). A total of 653 and 422 DEGs were found in M2 macrophages from HS1 and HS2 patients when compared to HC samples, several of the genes shown in Fig. 2h. The full list of DEGs can be found in Supplemental Table 4.

### H Syndrome as an Autoinflammatory Disease

**Fig. 3:**
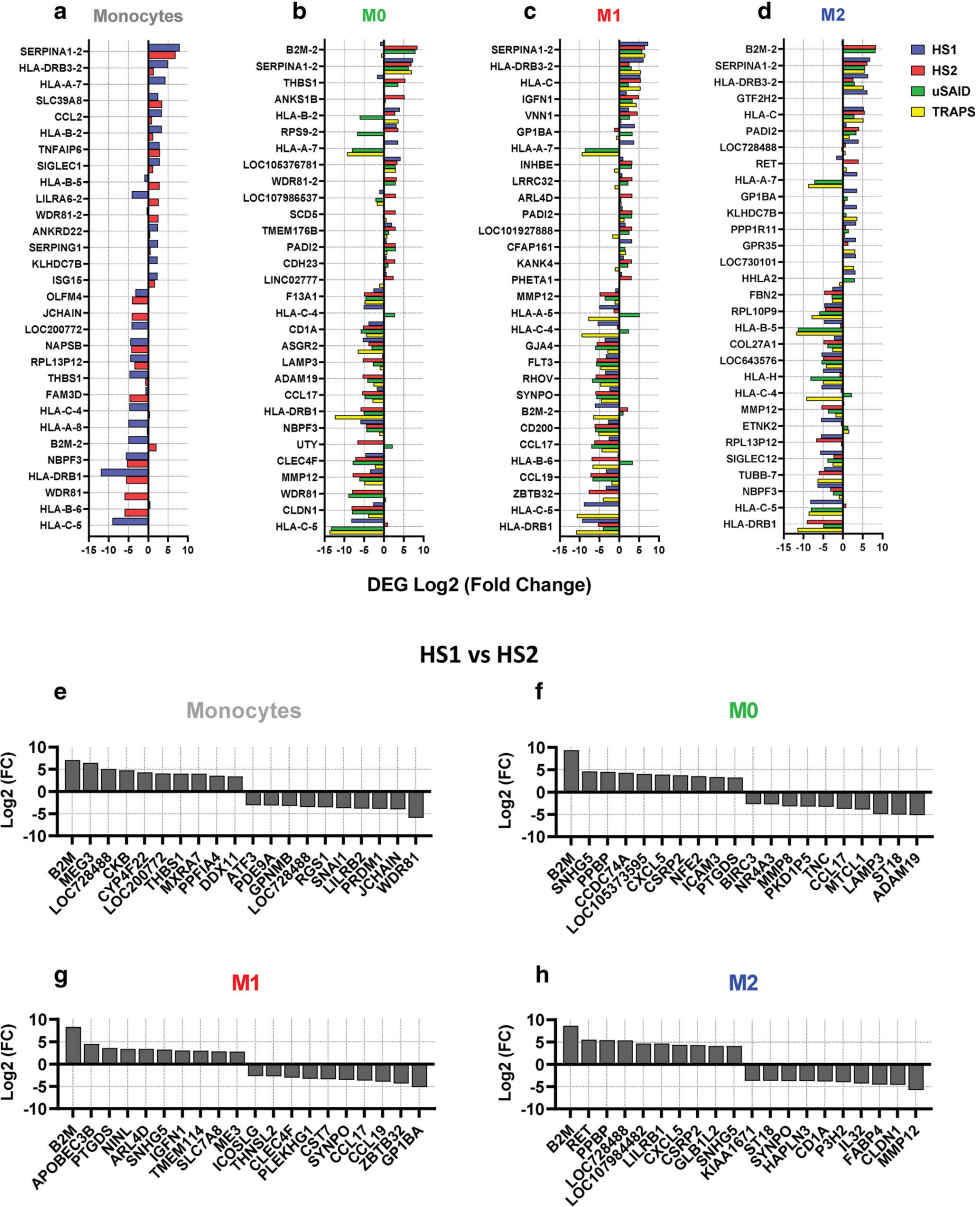
Signature DEGs in monocytes and macrophages from HS and SAID patients. Pairwise comparison of HS1, HS2, uSAID, and TRAPS patients vs HC (1–5) samples, in monocytes, M0, M1, and M2. (**a**–d) The top 15 upregulated and 15 downregulated DEGs, in each cell type, are shown in Log2 (fold change) based on the expression profile of the HS patients and matching the TRAPS patients to those DEGs. (**e**–**h**) Pairwise comparison of HS1 vs HS2, in monocytes, M0, M1, and M2. The top 10 upregulated and 10 downregulated DEGs, in each cell type, are shown in Log2 (fold change)

To classify genes with the most significant differences in the patients, the top 15 upregulated and 15 downregulated DEGs (ranked by fold change) in each cell type were identified (Fig. 3a–d). Additionally, to determine whether the gene expression profile of H syndrome patients resembles that of patients with SAID, we included the data from two SAID patients, which were also characterized by transcriptomic profiling alongside the HS patients (Fig. 1b–d; Supplemental Fig. 1A-D).

The *SERPINA1* gene, which encodes alpha1-antitrypsin (AAT), was found to be the most or second most upregulated gene in all cell types from both HS and SAID patients compared to HC (Fig. 3a–d). M0 macrophages from HS and SAID patients exhibited a comparable gene expression profile with similar levels of *SERPINA1*, *LOC105376781*, *F13A1*, *CD1A*, *ASGR2*, *CCL17*, *MMP12,* and *CLDN1* when compared to controls (Fig. 3b). Moreover, in M1 macrophages, 12 genes were found to be similarly expressed among the two HS and SAID patients, with *SERPINA1* being the top upregulated gene (Fig. 3c).

To further elucidate the unique DEGs, in monocytes and macrophages, which may be responsible for the autoinflammatory phenotype presented by HS2 patient, we compared the transcriptomic profile HS2 against HS1 and ranked the DEGs by fold change (Fig. 3e–h). Corroborating our previous findings, *SERPINA1* was not found in the list of DEGs, when comparing the two HS patients, suggesting that this gene is a shared marker in SAIDs. Remarkably, *B2M* was the top upregulated gene in all cell types from HS2 patient (Fig. 3e–h). M1 macrophages from HS2 patient showed increased levels of *NINL* and *SNHG5*, which have been associated with Hodgkin’s lymphoma lymphocytic-histiocytic predominance (Fig. 3g). In the M0 macrophages from the HS2 patient, we observed downregulation of *LAMP3,* which is related to lysosomal function.

**Fig. 4:**
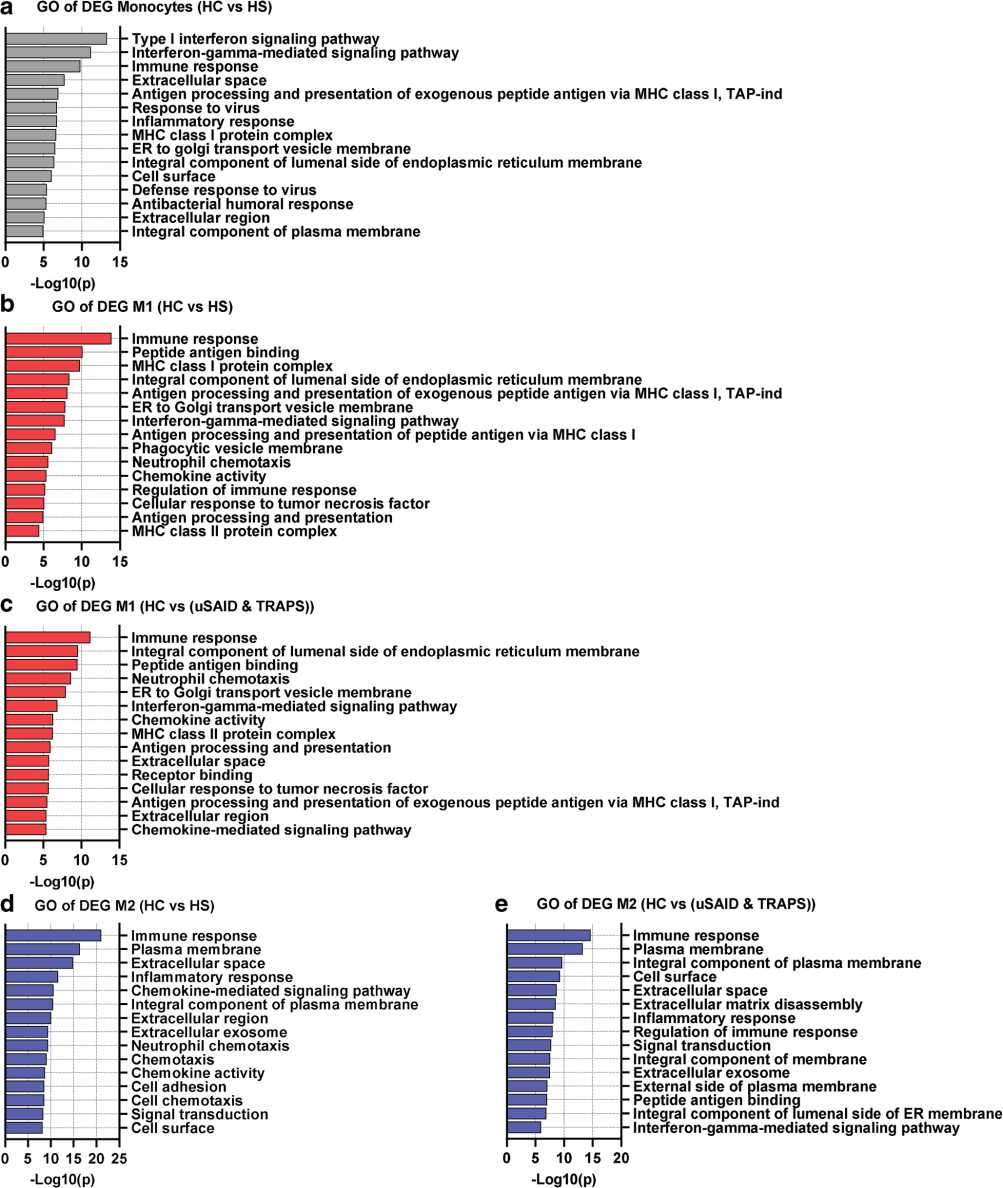
GO-based gene enrichment analyses on DEGs from HS and SAID patients. (**a**–**e**) Gene ontology (GO) enrichment analysis of all the DEGs in the HS and SAID patients, when compared to the HC (1–5) samples for each different cell type. The GO annotations correspond to the three major categories: biological process (BP), cellular compartment (CC), and molecular function (MF). The top 15 most significant GO terms (*p* < 0.01) are shown in descending order. Enrichment for GO terms was calculated using DAVID bioinformatics web source tools. The full list with all the terms can be found in Supplementary Table 2

We next performed gene ontology (GO) analysis of the DEGs in HS and SAIDs, when compared to HC. Macrophages from HS patients revealed a significant enrichment of GO terms related to immune responses, IFN signaling, and MHC class I (Fig. 4) (Supplemental Fig. 2A). For instance, the biological process (BP) GO term “Interferon-gamma-mediated signaling pathway” (GO:0060333) displayed a fold enrichment of 27.4 in monocytes, 14.1 in M0, 15.2 in M1, and 5.6 in M2, in both HS patients with FDR < 0.001 (Supplemental Table 5). Several of the top 15 GO terms in HS patients were also shown in uSAID and TRAPS patients (Fig. 4). For example, the GO term “Immune response” (GO:0006955) was in the top 4 terms from all cell types in HS and SAID patients (Fig. 4) (Supplemental Fig. 2A). Although the GO term “Inflammatory response” (GO:0006954) was significantly enriched in all the patients, this term was more enriched in M2 macrophages from all the patients, fold enrichment of 3.0 (HS) and 2.5 (uSAID and TRAPS) with a FDR < 0.0001 (Fig. 4d–e) (Supplemental Table 5).

**Fig. 5:**
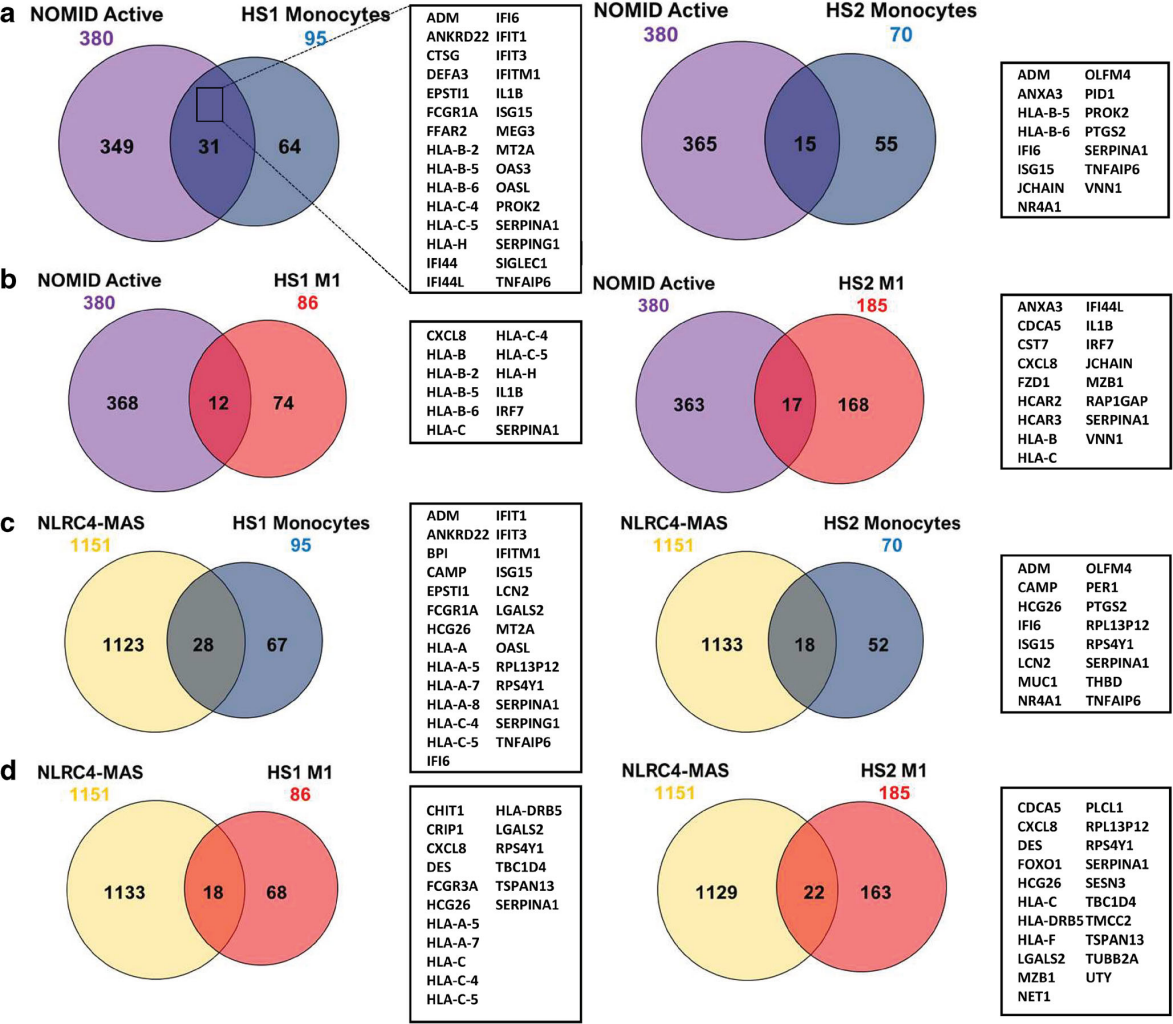

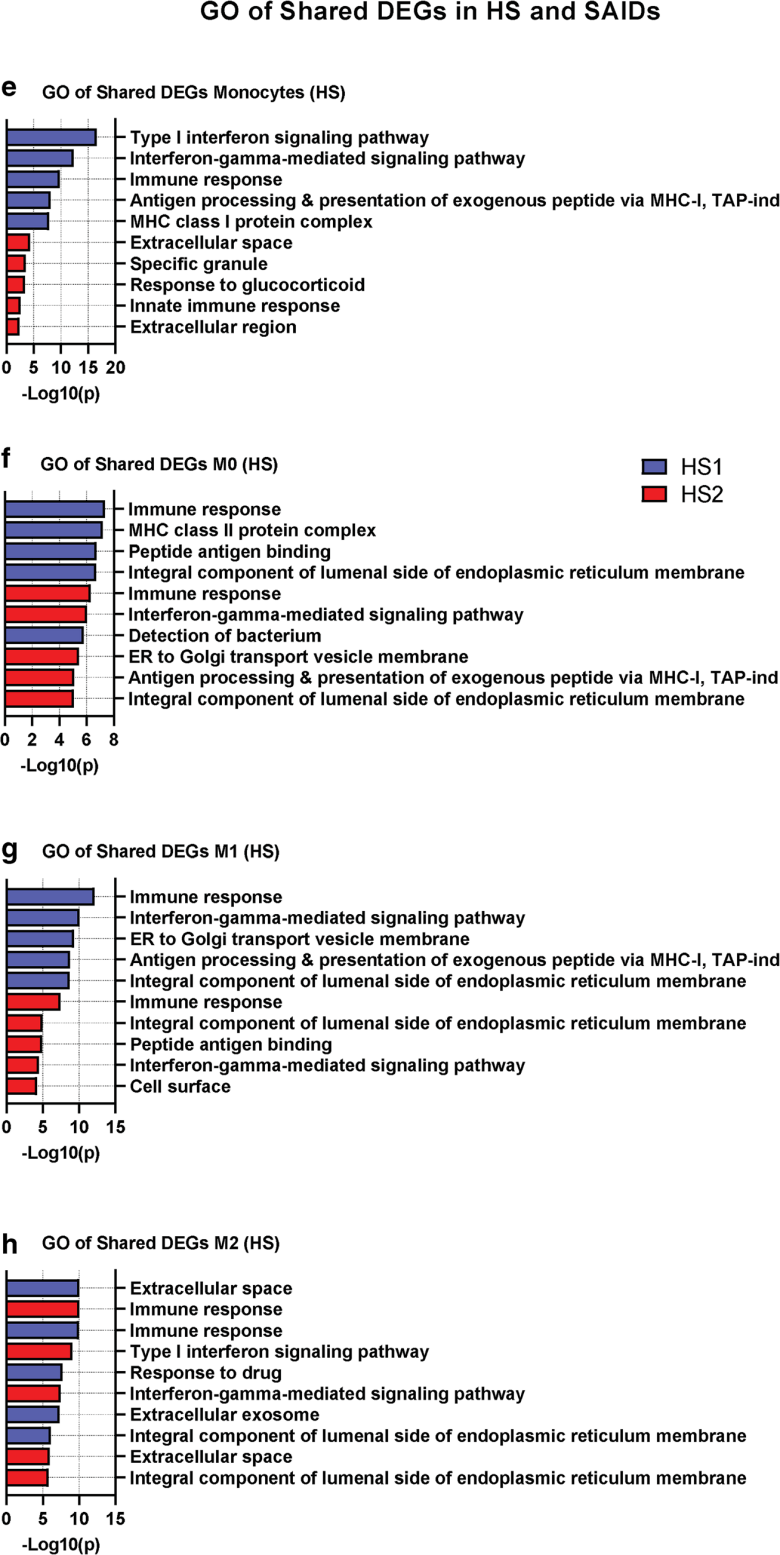
Shared DEGs and GO-based enrichment analyses in HS and SAIDs patients. (**a**–**d**) The number of DEGs in monocytes and M1 macrophages from HS1 (left) and HS2 (right) patients, NOMID Active (**a**–**b**), and NLRC4-MAS (**c**–**d**). The Venn diagrams show shared and unique DEGs for each group, when compared with their respective controls. The box on the right hand of each Venn diagram shows the shared DEGs in the groups. (**e**–**h**) List of GO enrichment terms in HS and SAIDs patients. DEGs were identified for NOMID active (active disease), NOMID inactive (after treatment), NLRC4-MAS, A20 stimulated (stimulated with TNF) and A20 unstimulated compared to the matched controls present in the same dataset and then compared to the DEGs identified in HS patients for each cell subtype. The top 5 terms for each HS patient are shown. GO terms were considered significant if *p* < 0.01. Further details of the SAIDs samples can be found in their respective studies [22, 23]

Furthermore, to compare gene expression profiles between HS patients and other defined SAID patients, we obtained and analyzed published transcriptome data from individuals diagnosed with either neonatal onset multisystem inflammatory disease (NOMID), NLRC4 macrophage activation syndrome (NLRC4-MAS) or A20 haploinsufficiency (due to mutation of *TNFAIP3*) [20, 21] (GSE57253 and GSE95078). DEGs were identified for each disorder, compared to the matched controls present in the same dataset and then compared to the DEGs identified in HS patients (Fig. 5). Consistent with our previous findings, the upregulation of *SERPINA1* was found to be shared in all SAID and HS patients’ samples (Fig. 5a–d). Moreover, the inflammatory markers CXCL8, TNFAIP6, and IL-1β were also shared between the conditions (Fig. 5a–d).

GO analysis of shared DEGs between HS and SAID patients revealed significant enrichment of GO terms, such as “type I interferon signaling pathway” (GO:0060337), “MHC class I protein complex” (GO:0042612) and “Immune response” (GO:0006955) (Fig. 5e–h) (Supplementary Table 3). Altogether, these data suggest a similar transcriptomic profile in HS and SAID patients.

### Sporadic and H Syndrome-Associated RDD

To determine if similar biological processes are involved in the pathogenesis of RDD related to H syndrome and in idiopathic cases, we compared transcriptomic profiles of tissue lymph node biopsies obtained from five sporadic cases of RDD and the HS2 patient. Pairwise comparison of biopsies from the five sporadic RDD cases against the HS2 failed to detect any DEGs suggesting that irrespective of the cause, there is a common pathological mechanism underpinning the development of RDD. To identify signaling pathways that might be associated with the pathogenesis of RDD, we filtered all the genes with fold change less than two and greater than − 2 and took the top 3000 genes with the highest expression. Panther pathway analysis revealed 27 significantly enriched pathways including “Inflammation mediated by chemokine and cytokine signaling pathway,” “JAK/STAT signaling pathway,” and “p38 MAPK pathway” (please see supplementary Tables 7 and 8 for full details).

### Dysregulated Cytokine Profile in HS and SAID Patients

**Fig. 6:**
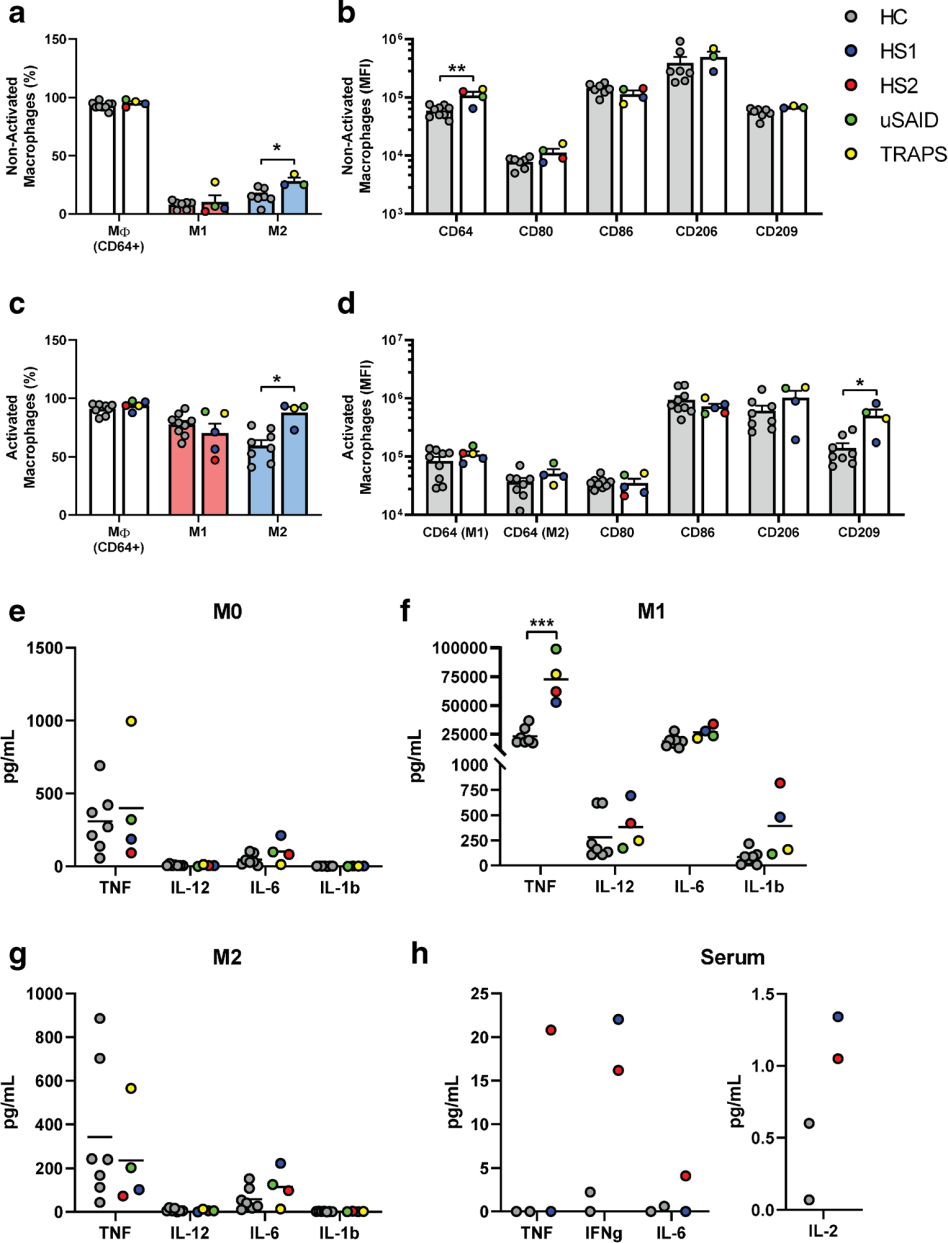
Dysregulated macrophage phenotype and cytokine profile in HS and SAID patients. M0 macrophages, polarized M1 or M2 macrophages were analyzed by flow cytometry; also, the cytokine profile of the macrophages was assessed using Luminex assay. (**a** and **b**) Represent M0 non-activated macrophages, where (**a**) show the percentage of macrophages (CD64^+^), M1 (CD64^+^, CD80^+^, CD86^+^), and M2 (CD64^+^, CD206^+^, CD209^+^) and (**b**) show the MFI of individual markers on each sample. (**c** and **d**) Represent activated macrophages, where (**c**) show the percentage of macrophages (CD64^+^), M1 (CD64^+^, CD80^+^, CD86^+^), and M2 (CD64^+^, CD206^+^, CD209^+^) and (**d**) show the MFI of individual markers on each sample. (**e**–**g**) Show the TNF, IL-12, IL-6, and IL1β cytokine levels of the M0, M1, and M2 macrophages on each sample. (**h**) Show the TNF, IFNγ, IL-6, and IL-2 cytokine levels on the serum of the two HS patients. Each symbol represents a unique individual sample for each of the patients and HC (1–9). In the patient samples, the two HS1 symbols represent independent experiments, in two different samples from the same subject. Statistical significance determined using the Holm-Sidak method; *p < 0.05, **p < 0.01, ***p < 0.001

Cellular plasticity confers macrophages the capacity to elicit different immune responses, pro-inflammatory (M1), or anti-inflammatory (M2), according to the environmental circumstances [22]. Abnormal proportions of M1 and M2 macrophages have also been reported in several immune disorders and are associated with disease progression [23]. We evaluated macrophage polarization in HS, uSAID, and TRAPS patients, where macrophages were classified as M1 (CD64^+^, CD80^+^, and CD86^+^) or M2 (CD64^+^, CD206^+^, and CD209^+^) according to their surface marker expression (Fig. 6). Prior to initiating differentiation, the TRAPS patient showed an increased number of M1 macrophages (27.3%); in contrast, the proportion of M2 macrophages was significantly increased in the uSAID, TRAPS, and HS1 patients compared to HC (Fig. 6a). Although proportionally no differences were observed in CD64 expression, the CD64 mean fluorescent intensity (MFI) was raised in all patients (Fig. 6a–b). When macrophages were activated, as described in Fig. 1a, HS patients showed a decrease in the proportion of M1 macrophages, which was more pronounced in HS2, and the proportion of M2 was significantly increased in the uSAID, TRAPS, and HS1 patients (Fig. 6c). CD80 MFI was slightly decreased in the two HS patients, while CD209 MFI was increased considerably in the two SAID and HS1 patients (Fig. 6d). The M1 and M2 gating strategy can be found in Supplemental Fig. 3. Cytokine profiles of patients presented some anomalies, showing high TNF in M0 macrophages from the TRAPS patient and significantly increased levels of TNF in M1 macrophages from all patients (Fig. 6e–f). Although M2 macrophages from the uSAID, TRAPS, and HS1 patients presented odd polarization ratios, no significant differences were observed in their cytokine levels (Fig. 6g). Moreover, increased serum levels of IFNγ, TNF, IL-6, and IL-2 were shown by HS2 patient, consistent with the patient’s inflammatory clinical features (Fig. 6h). Interestingly, both HS patients showed increased levels of IFNγ and IL-2 (Fig. 6h).

## Discussion

Systemic inflammation and development of RDD are common, yet unexplained, features in HS patients. Both remain difficult complications to manage since there are no standards of consistently effective treatment approaches. Our study was focused on investigating the potential role of monocytes and macrophages in this disease setting. Using the transcriptional approach, we provide additional data related to differentiation of monocytes into M0, M1, and M2 macrophages under ex vivo experimental condition. More importantly, this study offers new insights into possible mechanisms leading to autoinflammatory phenotype and RDD associated with H syndrome.

When we compared HS patients with and without inflammatory complications, there were some apparent differences at the transcriptomic level between HS1 and HS2, demonstrated by several DEGs, such as *ADAM19*, *CCL17*, *CCL19*, *CXCL5*, *IL32*, *MEG3*, *MMP12,* and *NINL.* Collectively these genes can control the cell migration in response to inflammatory stimuli [24, 25] and induce an inflammatory response [26–28]. All of these actions may account for the development of the autoinflammatory phenotype and RDD. Intriguingly, *B2M* was the top transcript upregulated in all cell subtypes from patient HS2. *B2M* is usually expressed by all cells in the human body, where the encoded protein forms complexes with HLA molecules [29]. Typically, increased levels of B2M are associated with increased cell turnover, as seen in lymphoproliferative conditions and chronic inflammation [30, 31]. In these disease states, high levels of B2M are associated with poor prognosis [31, 32], development of kidney failure [33] and secondary amyloidosis [34, 35]. B2M has not been routinely measured in patients with HS, so the risk of such complication in this patient group is unknown.

HS and SAID patients showed an overall similar transcriptomic and cytokine profile, with several GO terms enriched in both diseases. Transcriptomic analyses from HS and SAIDs from different datasets revealed overexpression of *SERPINA1*, which was shared in all cell subtypes. This finding might indicate a normal biological response towards an exaggerated inflammation, as ATT is induced upon inflammation and can modulate inflammation by inhibiting IL-8 and TNF [36–39]. This is consistent with our cytokine data, as we observed significantly higher levels of TNF in M1 macrophages from all patients. Moreover, H syndrome patients presented increased serum levels of IFNγ and GO enrichment of IFNγ-mediated signaling pathways. This finding may help to explain the abnormal activation of histiocytes in this condition and the increased levels of HLA genes, as IFNγ is known to induce HLA-I and HLA-II and inflammatory cytokines [40–42].

We also noted that the type I IFN pathway was enriched in both HS and SAID. Enhanced type I IFN signaling is typically associated with a group of autoinflammatory disorders termed type I interferonopathies [43–46]. However, it has recently been argued that the purely cytokine-/pathway-based approach towards classification and understanding of the pathogenesis of autoinflammatory disorders is probably an oversimplification [43–46]. There is likely to be an overlap between the biological processes which play a part in the pathogenesis of these disorders, and this might depend on the type of cell which has been studied and the stage of disease evolution.

When we compared transcriptomic profiles of tissue biopsies from HS2 and patients with sporadic forms of RDD, there were no apparent differences to report. This suggests that irrespective of the initial trigger for the development of RDD, there are common immunopathological abnormalities that drive this process.

Based on the transcriptomic and cytokine data, there are several potential treatment targets to consider. IFNγ has an essential effect on macrophage activation and clearly a pathogenic role in MAS considering that Emapalumab, which is an anti-IFNγ monoclonal antibody, has been recently approved for the treatment of hemophagocytic lymphohistiocytosis [47–49]. Furthermore, IFNγ has a role in MAPK activation [42], a pathway that has been successfully being targeted using MEK inhibitors, which have been shown to be efficacious in patients with histiocytic neoplasms [50].

In addition to IFNγ, we also found IL-1β, a prototypic pro-inflammatory cytokine associated with SAID, to be to be upregulated in HS patients. This was found in the M1 macrophages of both HS patients at not only the transcriptomic level but also at protein level. However, the role of IL-1β in the pathogenesis of autoinflammatory complications is probably complex. Although only HS2 patient presented with autoinflammatory complications, our findings suggest that low-grade chronic inflammation is a persistent feature in these patients and that other factors play a role in exacerbating this state leading it to become overtly pathogenic over time.

IL-2 is a pleiotropic cytokine which has been recognized for its role in the regulation and proliferation of effector T cells and regulatory T cells (Treg) [51, 52]. Studies have shown that low doses of IL-2 promote Treg development leading to amelioration of some autoimmune disorders [51, 52]. Nevertheless, it has been shown that systemic IL-2 administration can also lead to a cytokine storm and activate mononuclear phagocytes into mature antigen presenting cells [53]. In other studies, it was shown that after activation with IL-2, a small subset of innate lymphoid cells, named ILC2, increase their IL-5 production leading to activation of M2 macrophages [54, 55]. Interestingly, we found that IL-2 levels were increased in the serum of both H syndrome patients with higher proportions of M2 in HS1. Certainly, this IL-2 imbalance can potentially disturb macrophage activation, and low doses of IL-2 inhibitors may be efficacious in controlling sporadic inflammation.

However, the transcriptomic and cytokine data are not always easy to interpret and translate into successful treatment strategies. For example, although TRAPS is associated with elevated TNF levels, targeting IL-1beta has been far more effective and safer strategy than selectively targeting the TNF [56, 57]. Similarly, despite our data showing that type I IFN and JAK/STAT pathways are implicated in inflammatory and RDD pathogenesis, pegylated IFN has been used successfully to treat selected cases [58].

There are several limitations to this study. They include the limited number of patients who were included and type of the cells that we studied. Both HS and genetically defined SAIDs remain rare conditions, and therefore it is not always possible to exactly match patients according to age, sex, and previous treatments. Although we included monocytes into our study, we did not analyze distinct subpopulations, such as classical (CD14^++^, CD16^−^), intermediate (CD14^++^, CD16^+^), and non-classical (CD14^+^, CD16^+^) monocytes, which are all capable of distinctive cytokine production and have different roles in the pathogenesis of inflammatory responses [59–61]. This analysis should be included in future studies since the non-classical monocyte subpopulation was previously recognized to be increased in one patient diagnosed with H syndrome who presented with a combination of an autoinflammatory condition and immunodeficiency [62].

In summary, we provide a novel dataset which can help to further study monocytes and macrophages in HS and SAID patients and distinguish several DEGs and GO enrich pathways that are shared in these conditions. Altogether, we show that HS resembles an autoinflammatory condition with similar transcriptomic and cytokine landscape with the one observed in SAIDs.

## Supplementary Information

ESM 1 (XLSX 67 kb)

ESM 2 (XLSX 342 kb)

ESM 3 (XLSX 45 kb)

ESM 4 (XLSX 758 kb)

ESM 5 (XLSX 14 kb)

ESM 6 (DOCX 542 kb)

ESM 7 (DOCX 549 kb)
