# Supplementary material for: Identification of Critical Transcriptomic Signaling Pathways in Patients with H Syndrome and Rosai-Dorfman Disease
Source: J Clin Immunol. 2020 Dec 7;41(2):441–57. doi: 10.1007/s10875-020-00932-1 (PMC7858559; doi:10.1007/s10875-020-00932-1)
Supplement: Supplementary file 6 — (DOCX 542 kb) [file 10875_2020_932_MOESM6_ESM.docx]

**Supplemental Data**

**Identification of critical transcriptomic signalling pathways in macrophages from H syndrome patients**

Samuel Lara-Reyna^1,2^, James A. Poulter^1,2^, Elton J.R. Vasconcelos^3^, Mark Kacar^1,6^, Michael F. McDermott^1^, Reuben Tooze^4^, Rainer Doffinger^5^, Sinisa Savic^1,6,^*

^1^Leeds Institute of Rheumatic and Musculoskeletal Medicine, University of Leeds, Leeds LS9 7TF, UK

^2^Leeds Institute of Medical Research, University of Leeds, Leeds, LS9 7TF, UK

^3^Leeds Omics, University of Leeds, Leeds LS2 9JT, UK

^4^Section of Experimental Haematology, Leeds Institute of Cancer and Pathology, University of Leeds, Leeds, UK

^5^Department of Clinical Biochemistry and Immunology, Addenbrooke's Hospital, Cambridge, CB2 2QQ, UK

^6^Department of Clinical Immunology and Allergy, St James's University Hospital, Leeds, LS9 7TF, UK

***Correspondence:**
Sinisa Savic
[S.Savic@leeds.ac.uk](mailto:S.Savic@leeds.ac.uk)

**Methods**

**Supplemental Table 1. Demographic data from lymph node biopsies**

| ID | Age (years) stated at the time when biopsy was taken | Sex | Source of material | Diagnosis |
| --- | --- | --- | --- | --- |
| T1 | 73 | M | LN | Idiopathic RDD |
| T2 | 30 | M | LN | Idiopathic RDD |
| T3 | 4 | F | LN | Idiopathic RDD |
| T4 | 19 | F | LN | HS and RDD |
| T5 | 78 | M | LN | Idiopathic RDD |
| T6 | 37 | F | LN | Idiopathic RDD |

M-male; F-female; LN-lymph node; RDD- Rosai-Dorfman disease

**Supplemental Table 2. List of reagents**

| **REAGENTS** | **SOURCE** | **IDENTIFIER** |
| --- | --- | --- |
| Antibodies | | |
| AF 700 Mouse Anti-Human CD64 | BD Biosciences | Cat# 561188; RRID: AB_10612007 |
| APC Anti-Human CD80 | Miltenyi Biotec | Cat# 130-117-719; RRID: AB_2751414 |
| PE-Cy7 Mouse Anti-Human CD86 | BD Biosciences | Cat# 561128; RRID: AB_10563077 |
| FITC Mouse Anti-Human CD206 | BD Biosciences | Cat# 551135; RRID: AB_394065 |
| V450 Mouse Anti-Human CD209 | BD Biosciences | Cat# 561275; RRID: AB_10694104 |
| Biological Samples | | |
| Human Blood Samples | St James's University Hospital |  |
| Chemicals, Peptides, and Recombinant Proteins | | |
| Lymphoprep | Axis Shield | Cat# 1114544 |
| EasySep Human Monocyte Isolation Kit | StemCell | Cat# 19359 |
| Recombinant Human GM-CSF | PeproTech | Cat# 300-03 |
| Recombinant Human IFN-γ | PeproTech | Cat# 300-02 |
| Recombinant Human IL-13 | PeproTech | Cat# 200-13 |
| Recombinant Human IL-4 | PeproTech | Cat# 200-04 |
| LPS | InvivoGen | Cat# tlrl-3pelps |
| TRIzol Reagent and Phasemaker Tubes Complete System | ThermoFisher Scientific | Cat# A33251 |
| Software | | |
| GraphPad Prism8 | Graphpad software | N/A |
| CytExpert Software | Beckman Coulter | N/A |
| Flow Jo Vx0.7 | FlowJo, LLC | N/A |

**Supplemental Table 3**

|  | **HS1** | | | |
| --- | --- | --- | --- | --- |
|  | **(SAID \| shared \| mono)** | **(SAID \| shared \| M0)** | **(SAID \| shared \| M1)** | **(SAID \| shared \| M2)** |
| **A20 Stimulated** | 5005 \| 39 \| 56 | 5011 \| 33 \| 62 | 5011 \| 33 \| 53 | 4759 \| 285 \| 417 |
| **A20 Unstim** | 4074 \| 40 \| 55 | 4089 \| 25 \| 70 | 4087 \| 27 \| 59 | 3873 \| 241 \| 461 |
| **NOMID Inactive** | 269 \| 12 \| 83 | 273 \| 8 \| 87 | 270 \| 11 \| 75 | 250 \| 31 \| 671 |
|  | **HS2** | | | |
|  | **(SAID \| shared \| mono)** | **(SAID \| shared \| M0)** | **(SAID \| shared \| M1)** | **(SAID \| shared \| M2)** |
| **A20 Stimulated** | 5013 \| 31 \| 39 | 4977 \| 67 \| 120 | 4973 \| 71 \| 114 | 4840 \| 204 \| 263 |
| **A20 Unstim** | 4087 \| 27 \| 43 | 4060 \| 54 \| 133 | 4054 \| 60 \| 125 | 3923 \| 191 \| 276 |
| **NOMID Inactive** | 274 \| 7 \| 63 | 269 \| 12 \| 175 | 269 \| 12 \| 173 | 250 \| 31 \| 436 |

Pairwise comparison of each SAIDs vs HS samples, in monocytes, M0, M1 and M2. Then the list of DEGs unique for the SAID, shared between the two conditions, or unique to the HS sample is shown in the table. DEGs were q < 0.05.

**Supplemental Figures**

**
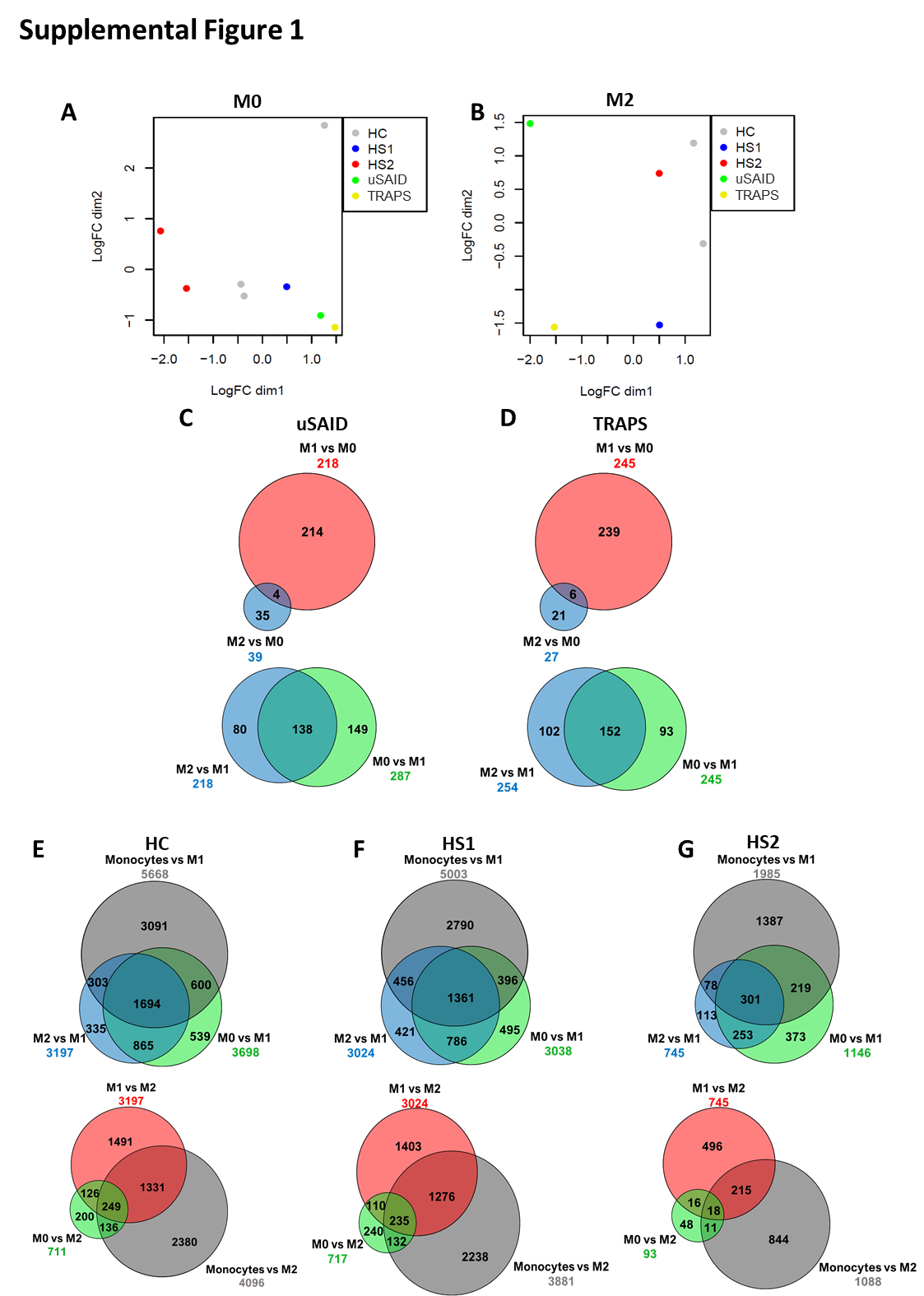
**

**Supplemental Figure 1**

**Complementary sample analysis in both monocytes and macrophages.** These figures complement Figure 1. (A and B) MDS plot showing the global transcriptomic profile in M0 and M2 of all the HC and patients' samples. (C-G) Number of differentially expressed genes (DEGs) in pairwise comparisons of the different cell types are given, where M0 (top) and M1 (bottom) are compared to each other cell type in the uSAID and TRAPS patients. The Venn diagrams show shared and unique DEGs for each cell type in, (C) uSAID and (D) TRAPS samples. Number of differentially expressed genes in pairwise comparisons of the different cell types are given, where M1 (top) and M2 (bottom) are compared to each other cell type in the HC and HS patients. The Venn diagrams show shared and unique DEGs for each cell type in, (E) HC, (F) HS1 and (G) HS2 samples.


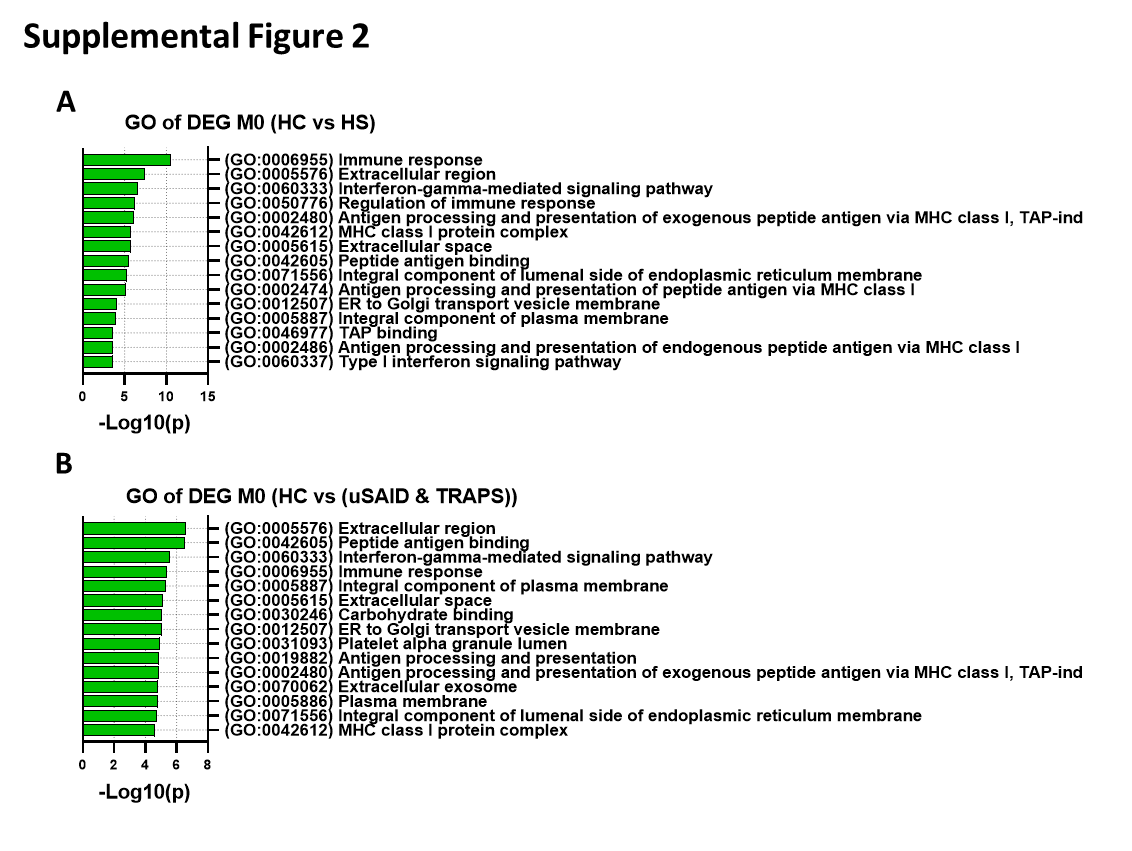


**Supplemental Figure 2**

**GO-based gene enrichment analyses on DEGs in M0 from HS, uSAID and TRAPS patients.** These figures complement Figure 5. (A and B) Gene ontology (GO) enrichment analysis of all the DEGs in the HS, uSAID and TRAPS patients, when compared to the HC samples for M0. The GO annotations correspond to the three major categories: Biological process (BP); Cellular compartment (CC); Molecular function (MF). The top 15 most significant GO terms (p < 0.01) are shown in descending order. Enrichment for GO terms was calculated using DAVID bioinformatics web source tools. The full list with all the terms can be found in Supplementary table 2.


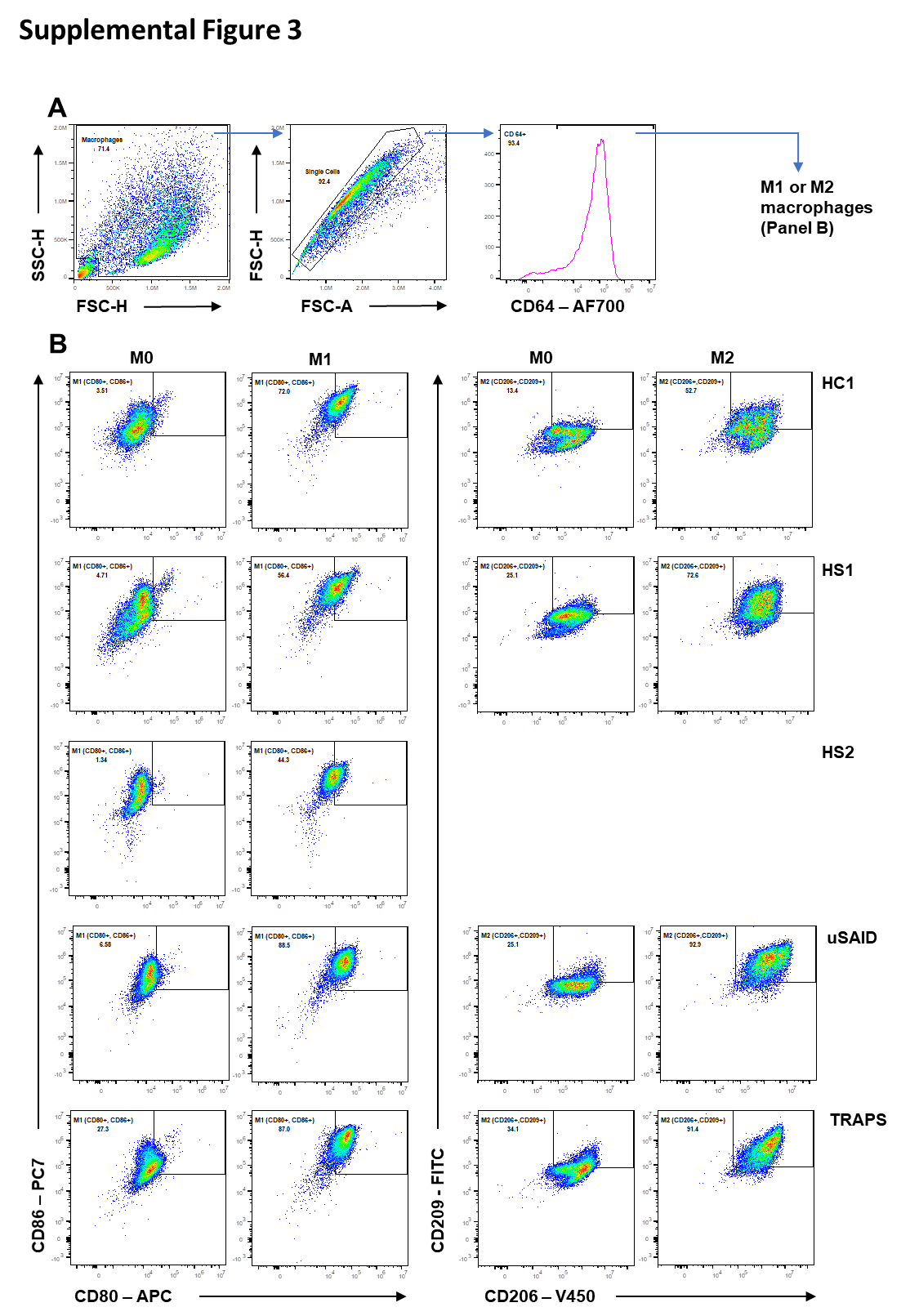


**Supplemental Figure 3**

**Macrophage flow cytometry gating strategy.** These figures complement Figure 4. Representative gating strategy for M0, M1 and M2 macrophages. Monocytes (CD14^+^) were isolated and cultured as described in the methods section. (A) Cells were positively selected based on size (SSC/FSC), then single cells were positively selected based (FSC-H/FSC-A) and finally cells positive for the marker CD64 were considered as macrophages. (B) CD64^+^ cells were considered to be M1 macrophages only when they were CD80^+^ and CD86^+^; whereas, cells CD206^+^ and CD209^+^ were considered to be M2 macrophages. All the antibodies are listed in detail in the resource table.

**Legends for Excel supplemental files**

**Supplemental Table 4**

**DEGs in monocytes and macrophages**. List of DEGs in HS, uSAID and TRAPS patients. The table is divided in DEGs in monocytes (Tab 1), M0 macrophages (Tab 2), M1 macrophages (Tab 3) and M2 macrophages (Tab 4), all DEGs were q < 0.05. The comparisons in each cell type were done independently of the other cell types.

**Supplemental Table 5**

**GO enrichment terms of the DEGs in monocytes and macrophages**. List of GO enrichment terms in HS, uSAID and TRAPS patients. The table is divided in GO terms, from the list of DEGs obtained when comparing; HC vs HS monocytes (Tab 1), HC vs HS M0 macrophages (Tab 2), HC vs HS M1 macrophages (Tab 3), HC vs HS M2 macrophages (Tab 4), HC vs (uSAID and TRAPS) M0 macrophages (Tab 5), HC vs (uSAID and TRAPS) M1 macrophages (Tab 6), HC vs (uSAID and TRAPS) M2 macrophages (Tab 7). GO terms were considered significant if p < 0.01.

**Supplemental Table 6**

**GO enrichment terms of the shared DEGs in HS and SAID patients**. List of GO enrichment terms in HS and SAID patients. DEGs were identified for NOMID active (active disease), NOMID inactive (after treatment), NLRC4-MAS, A20 stimulated (stimulated with TNF) and A20 unstimulated compared to the matched controls present in the same dataset, and then compared to DEGs identified in HS patients for each cell subtype. Shared DEGs in each comparison are shown as indicated next. The table is divided in GO terms, from the list of shared DEGs obtained when comparing the SAIDs vs; HS1 monocytes (Tab 1), HS2 monocytes (Tab 2), HS1 M0 macrophages (Tab 3), HS2 M0 macrophages (Tab 4), HS1 M1 macrophages (Tab 5), HS2 M1 macrophages (Tab 6), HS1 M2 macrophages (Tab 7) and HS2 M2 macrophages (Tab 8). GO terms were considered significant if p < 0.01. Further details of the SAIDs samples can be found in their respective studies [22, 23].

**Supplemental Table 7**

**Gene expression in RDD and HS2 tissue biopsies**. List of gene expression in five lymph node tissue biopsies from RDD compared to the HS2 patient. The table shows all the gene names, values and corrected p values from the five RDD together against the HS2. No DEGs were detected. All the tests were perform as described in the methods section.

**Supplemental Table 8**

**Panther pathway analysis of similarly expressed genes in RDD and HS2 tissue biopsies.** All the genes from the comparison in Supplemental Table 5 were filtered and only genes with fold change value less than 2 and greater than -2 were considered for the Panther pathway analysis. The top 3,000 genes with the highest expression were used to produce this table. Only terms with p < 0.05 were considered.
