# Supplementary material for: Identification of Critical Transcriptomic Signaling Pathways in Patients with H Syndrome and Rosai-Dorfman Disease
Source: J Clin Immunol. 2020 Dec 7;41(2):441–57. doi: 10.1007/s10875-020-00932-1 (PMC7858559; doi:10.1007/s10875-020-00932-1)
Supplement: Supplementary file 7 — (DOCX 549 kb) [file 10875_2020_932_MOESM7_ESM.docx]

**Supplemental Data**

**Identification of critical transcriptomic signalling pathways in macrophages from H syndrome patients**

Samuel Lara-Reyna^1,2^, James A. Poulter^1,2^, Elton J.R. Vasconcelos^3^, Mark Kacar^1,6^, Michael F. McDermott^1^, Reuben Tooze^4^, Rainer Doffinger^5^, Sinisa Savic^1,6,^*

^1^Leeds Institute of Rheumatic and Musculoskeletal Medicine, University of Leeds, Leeds LS9 7TF, UK

^2^Leeds Institute of Medical Research, University of Leeds, Leeds, LS9 7TF, UK

^3^Leeds Omics, University of Leeds, Leeds LS2 9JT, UK

^4^Section of Experimental Haematology, Leeds Institute of Cancer and Pathology, University of Leeds, Leeds, UK

^5^Department of Clinical Biochemistry and Immunology, Addenbrooke's Hospital, Cambridge, CB2 2QQ, UK

^6^Department of Clinical Immunology and Allergy, St James's University Hospital, Leeds, LS9 7TF, UK

***Correspondence:**
Sinisa Savic
[S.Savic@leeds.ac.uk](mailto:S.Savic@leeds.ac.uk)

**Methods**

**Clinical and demographic data from Lymph node biopsies**

Tissue biopsy 1 (T1)

This sample is from a 73-year-old (at the time of collection) male patient. This lymph node shows general preservation of normal architecture. There are expanded B cell follicles and some of these contain germinal centres. The main feature of the lymph node is the expansion of the sinuses; these contain a significant proportion of large S100^+^ macrophages many of which are phagocytic. These cells contain red cells and lymphocytes. A very marked reactive plasmacytosis is present. This lymph node shows an unusual reactive pattern but some of the features are typically seen in Rosai-Dorfman disease (RDD).

Tissue biopsy 2 (T2)

This sample is from a 30-year-old (at the time of collection) male patient. In the lymph node, the capsule in places shows marked thickening. Overall architecture is generally preserved. There are numerous small B cell follicles. The most striking feature, however, is that of sinus histiocytosis. A number of the histiocytes are actively phagocytosing lymphocytes, plasma cells and granulocytes. There is a striking increase in plasma cells in addition. The lymph node shows features of RDD.

Tissue biopsy 3 (T3)

This sample is from a 4-year-old (at the time of collection) female patient. This is a greatly enlarged lymph node with general preservation of the nodal architecture. The main feature is the presence of expanded sinuses. These contain a population of large macrophages with open nuclei and prominent nucleoli. These cells show lymphocyte phagocytosis with strong expression of S100. This is sinus histiocytosis with massive lymphadenopathy RDD.

Tissue biopsy 4 (T4)

This sample is from a 19-year-old (at the time of collection) female patient. This patient is the one described in the main manuscript as HS2. The overall architecture of the lymph node is preserved. There is follicular and paracortical expansion with plasmacytosis. There is replacement of the nodal sinuses by a population of large S100+ histiocytes many of which contain lymphocytes and plasma cells. The morphological features are those of RDD (sinus histiocytosis with massive lymphadenopathy).

Tissue biopsy 5 (T5)

This sample is from a 78-year-old (at the time of collection) male patient. The nodes are enlarged and show an extensive proliferation of plasma cells in the medulla these are polytypic with both kappa and lambda positive cells present. Follicles show hyalinised germinal centres with onionskin pattern to follicular mantles. In addition, sinuses are expended and populated by large macrophages with open vesicular nuclei an ample cytoplasm showing evidence of emperipolesis. These cells are S100^+^ and CD1a^-^. This is an unusual combination of features; the plasma cells and follicular morphology are consistent with a plasma cell variant of Castleman's disease. On the other hand, the sinus macrophages have the typical features of RDD.

Tissue biopsy 6 (T6)

This sample is from a 37-year-old (at the time of collection) female patient. The enlarged lymph node shows a densely fibrotic capsule along with follicular hyperplasia. B cell follicles appear reactive as they contain a tangible body of macrophages and lack BCL2 protein expression. An interfollicular B cell expansion is noted along with scattered CD30^+^ cells. There are Touton type giant cells and focal collagen degeneration with a vague granulomatous pattern and accompanying plasma cells and lymphocytes. In addition, there is a population of plump histiocytic/macrophage lineage cells extending into fat. This population of macrophage lineage cells is positive for PU.1, CD68, CD163 and S100 and lacks expression of CD1a and Langerin. Focally there is a suggestion of emperipolesis. The pattern is unusual and raises the differential diagnosis of extra-nodal RDD. While the Touton type giant cells in fibrotic background might also be in keeping with Erdheim Chester disease the S100^+^ phenotype is not typical studies for BRAF and MAP2K1 mutations have failed on this sample. Extra-nodal RDD is therefore the more likely potential diagnosis in this case.

**Table of reagents**

| **REAGENTS** | **SOURCE** | **IDENTIFIER** |
| --- | --- | --- |
| Antibodies | | |
| AF 700 Mouse Anti-Human CD64 | BD Biosciences | Cat# 561188; RRID: AB_10612007 |
| APC Anti-Human CD80 | Miltenyi Biotec | Cat# 130-117-719; RRID: AB_2751414 |
| PE-Cy7 Mouse Anti-Human CD86 | BD Biosciences | Cat# 561128; RRID: AB_10563077 |
| FITC Mouse Anti-Human CD206 | BD Biosciences | Cat# 551135; RRID: AB_394065 |
| V450 Mouse Anti-Human CD209 | BD Biosciences | Cat# 561275; RRID: AB_10694104 |
| Biological Samples | | |
| Human Blood Samples | St James's University Hospital |  |
| Chemicals, Peptides, and Recombinant Proteins | | |
| Lymphoprep | Axis Shield | Cat# 1114544 |
| EasySep Human Monocyte Isolation Kit | StemCell | Cat# 19359 |
| Recombinant Human GM-CSF | PeproTech | Cat# 300-03 |
| Recombinant Human IFN-γ | PeproTech | Cat# 300-02 |
| Recombinant Human IL-13 | PeproTech | Cat# 200-13 |
| Recombinant Human IL-4 | PeproTech | Cat# 200-04 |
| LPS | InvivoGen | Cat# tlrl-3pelps |
| TRIzol Reagent and Phasemaker Tubes Complete System | ThermoFisher Scientific | Cat# A33251 |
| Software | | |
| GraphPad Prism8 | Graphpad software | N/A |
| CytExpert Software | Beckman Coulter | N/A |
| Flow Jo Vx0.7 | FlowJo, LLC | N/A |

**Supplemental Tables**

**Supplemental Table 3**

|  | **HS1** | | | |
| --- | --- | --- | --- | --- |
|  | **(SAID \| shared \| mono)** | **(SAID \| shared \| M0)** | **(SAID \| shared \| M1)** | **(SAID \| shared \| M2)** |
| **A20 Stimulated** | 5005 \| 39 \| 56 | 5011 \| 33 \| 62 | 5011 \| 33 \| 53 | 4759 \| 285 \| 417 |
| **A20 Unstim** | 4074 \| 40 \| 55 | 4089 \| 25 \| 70 | 4087 \| 27 \| 59 | 3873 \| 241 \| 461 |
| **NOMID Inactive** | 269 \| 12 \| 83 | 273 \| 8 \| 87 | 270 \| 11 \| 75 | 250 \| 31 \| 671 |
|  | **HS2** | | | |
|  | **(SAID \| shared \| mono)** | **(SAID \| shared \| M0)** | **(SAID \| shared \| M1)** | **(SAID \| shared \| M2)** |
| **A20 Stimulated** | 5013 \| 31 \| 39 | 4977 \| 67 \| 120 | 4973 \| 71 \| 114 | 4840 \| 204 \| 263 |
| **A20 Unstim** | 4087 \| 27 \| 43 | 4060 \| 54 \| 133 | 4054 \| 60 \| 125 | 3923 \| 191 \| 276 |
| **NOMID Inactive** | 274 \| 7 \| 63 | 269 \| 12 \| 175 | 269 \| 12 \| 173 | 250 \| 31 \| 436 |

Pairwise comparison of each SAIDs vs HS samples, in monocytes, M0, M1 and M2. Then the list of DEGs unique for the SAID, shared between the two conditions, or unique to the HS sample is shown in the table. DEGs were q < 0.05.

**Supplemental Figures**

**
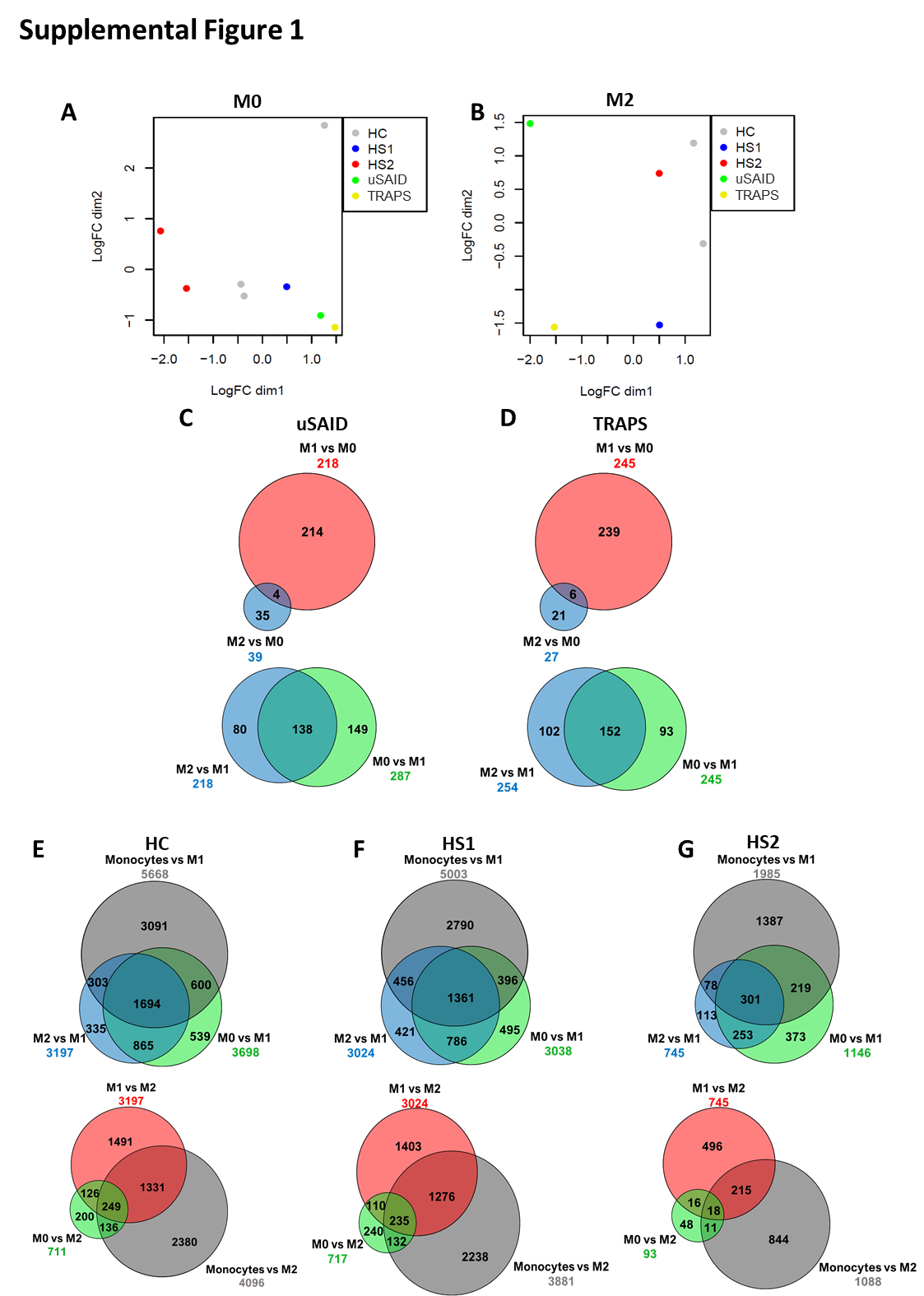
**

**Supplemental Figure 1**

**Complementary sample analysis in both monocytes and macrophages.** These figures complement Figure 1. (A and B) MDS plot showing the global transcriptomic profile in M0 and M2 of all the HC and patients' samples. (C-G) Number of differentially expressed genes (DEGs) in pairwise comparisons of the different cell types are given, where M0 (top) and M1 (bottom) are compared to each other cell type in the uSAID and TRAPS patients. The Venn diagrams show shared and unique DEGs for each cell type in, (C) uSAID and (D) TRAPS samples. Number of differentially expressed genes in pairwise comparisons of the different cell types are given, where M1 (top) and M2 (bottom) are compared to each other cell type in the HC and HS patients. The Venn diagrams show shared and unique DEGs for each cell type in, (E) HC, (F) HS1 and (G) HS2 samples.


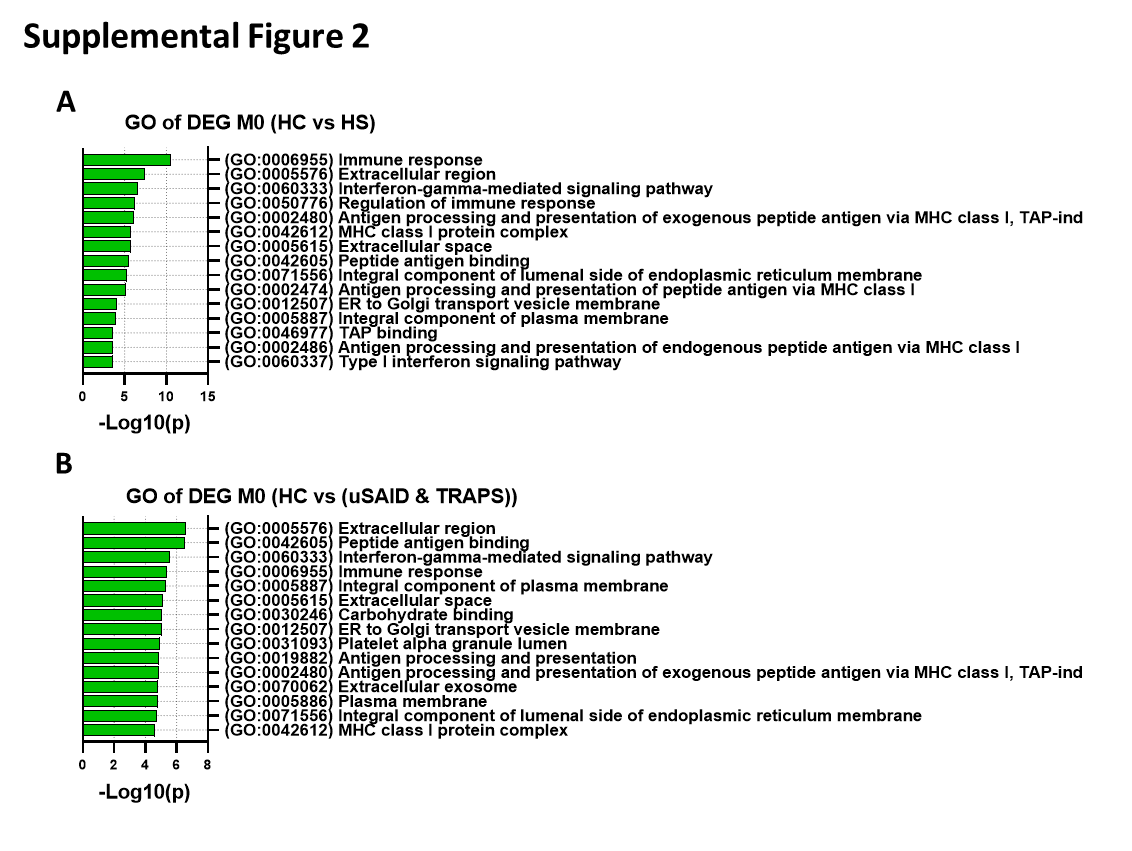


**Supplemental Figure 2**

**GO-based gene enrichment analyses on DEGs in M0 from HS, uSAID and TRAPS patients.** These figures complement Figure 5. (A and B) Gene ontology (GO) enrichment analysis of all the DEGs in the HS, uSAID and TRAPS patients, when compared to the HC samples for M0. The GO annotations correspond to the three major categories: Biological process (BP); Cellular compartment (CC); Molecular function (MF). The top 15 most significant GO terms (p < 0.01) are shown in descending order. Enrichment for GO terms was calculated using DAVID bioinformatics web source tools. The full list with all the terms can be found in Supplementary table 2.


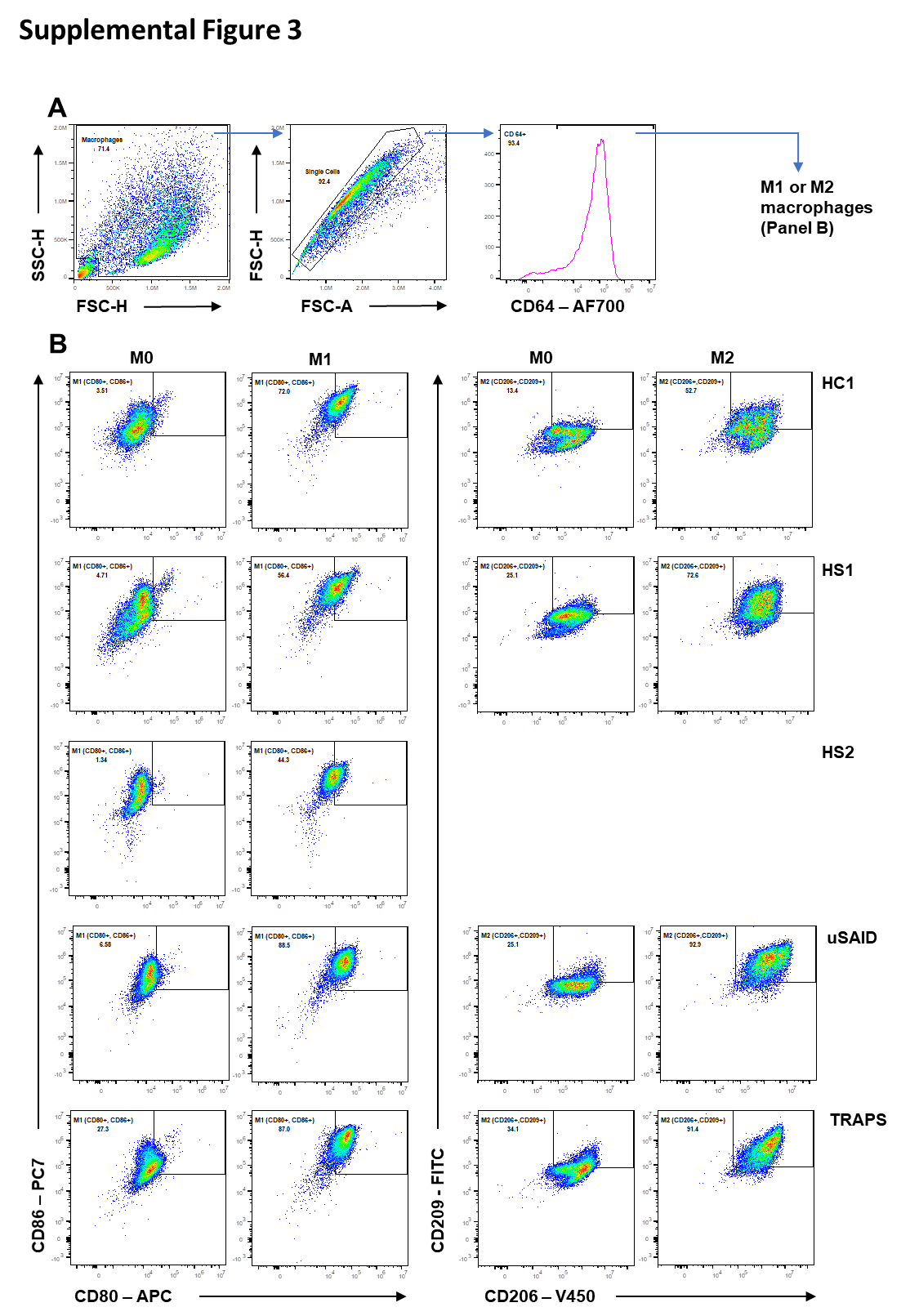


**Supplemental Figure 3**

**Macrophage flow cytometry gating strategy.** These figures complement Figure 4. Representative gating strategy for M0, M1 and M2 macrophages. Monocytes (CD14^+^) were isolated and cultured as described in the methods section. (A) Cells were positively selected based on size (SSC/FSC), then single cells were positively selected based (FSC-H/FSC-A) and finally cells positive for the marker CD64 were considered as macrophages. (B) CD64^+^ cells were considered to be M1 macrophages only when they were CD80^+^ and CD86^+^; whereas, cells CD206^+^ and CD209^+^ were considered to be M2 macrophages. All the antibodies are listed in detail in the resource table.

**Legends for Excel supplemental files**

**Supplemental Table 1**

**DEGs in monocytes and macrophages**. List of DEGs in HS, uSAID and TRAPS patients. The table is divided in DEGs in monocytes (Tab 1), M0 macrophages (Tab 2), M1 macrophages (Tab 3) and M2 macrophages (Tab 4), all DEGs were q < 0.05. The comparisons in each cell type were done independently of the other cell types.

**Supplemental Table 2**

**GO enrichment terms of the DEGs in monocytes and macrophages**. List of GO enrichment terms in HS, uSAID and TRAPS patients. The table is divided in GO terms, from the list of DEGs obtained when comparing; HC vs HS monocytes (Tab 1), HC vs HS M0 macrophages (Tab 2), HC vs HS M1 macrophages (Tab 3), HC vs HS M2 macrophages (Tab 4), HC vs (uSAID and TRAPS) M0 macrophages (Tab 5), HC vs (uSAID and TRAPS) M1 macrophages (Tab 6), HC vs (uSAID and TRAPS) M2 macrophages (Tab 7). GO terms were considered significant if p < 0.01.

**Supplemental Table 4**

**GO enrichment terms of the shared DEGs in HS and SAID patients**. List of GO enrichment terms in HS and SAID patients. DEGs were identified for NOMID active (active disease), NOMID inactive (after treatment), NLRC4-MAS, A20 stimulated (stimulated with TNF) and A20 unstimulated compared to the matched controls present in the same dataset, and then compared to DEGs identified in HS patients for each cell subtype. Shared DEGs in each comparison are shown as indicated next. The table is divided in GO terms, from the list of shared DEGs obtained when comparing the SAIDs vs; HS1 monocytes (Tab 1), HS2 monocytes (Tab 2), HS1 M0 macrophages (Tab 3), HS2 M0 macrophages (Tab 4), HS1 M1 macrophages (Tab 5), HS2 M1 macrophages (Tab 6), HS1 M2 macrophages (Tab 7) and HS2 M2 macrophages (Tab 8). GO terms were considered significant if p < 0.01. Further details of the SAIDs samples can be found in their respective studies [22, 23].
